# Belimumab-driven reductions in retinal microvascular density assessed by optical coherence tomography angiography: insights from systemic lupus erythematosus patients

**DOI:** 10.3389/fimmu.2025.1511133

**Published:** 2025-06-16

**Authors:** Maierhaba Maitiyaer, Jingyu Zhang, Peiyi Li, Dingwen Jiang, Huangdong Li, Zeying Lin, Ziguan Ye, Yongbao Huo, Wenhui Huang, Li Wang, Zhiping Liu, Shuilian Yu

**Affiliations:** ^1^ Department of Rheumatology, The Second Affiliated Hospital, Guangzhou Medical University, Guangzhou, Guangdong, China; ^2^ Ophthalmic Center, The Second Affiliated Hospital, Guangzhou Medical University, Guangzhou, Guangdong, China; ^3^ Department of Rheumatology, Liwan Central Hospital of Guangzhou, Guangzhou, Guangdong, China; ^4^ Department of Clinical Medicine, The Second School of Clinical Medicine, Guangzhou Medical University, Guangzhou, Guangdong, China

**Keywords:** systemic lupus erythematosus, lupus nephritis, optical coherence tomography angiography, retinal microvascular, Belimumab

## Abstract

**Background:**

Systemic lupus erythematosus (SLE) and lupus nephritis (LN) are associated with retinal microvascular changes that may reflect disease severity. This study aimed to evaluate differences in retinal vascular density (VD) between SLE patients with and without LN and assess the impact of rheumatological treatments on VD.

**Methods:**

A cross-sectional study was conducted with 54 SLE patients (21 with LN, 33 without LN). Retinal VD was measured using optical coherence tomography angiography (OCTA), focusing on superficial and deep capillary plexus VD (SCP-VD and DCP-VD). The impact of Belimumab and other treatments was analyzed. Linear regression assessed the effects of LN status and treatments on DCP parafoveal VD. Lymphocyte subsets and cytokines were compared before and after Belimumab treatment.

**Result:**

LN patients showed significantly reduced macular vascular density compared with non-LN patients. Belimumab treatment (≥8 times) and hydroxychloroquine use (>5 years) were independently associated with lower DCP-VD, particularly in parafoveal areas. Cumulative doses of HCQ and glucocorticoids negatively correlated with VD. Linear regression showed a significant negative association between Belimumab treatment and parafoveal DCP-VD. Notably, Belimumab treatment led to reductions in serum CD19+ B cells and IL-10 levels.

**Conclusions:**

LN patients demonstrated distinct retinal microvascular alterations. Long-term Belimumab and HCQ treatments were associated with decreased retinal VD. Regular retinal health monitoring was recommended to prevent microvascular complications in SLE patients undergoing prolonged treatment.

## 1 Introduction

Ocular manifestations are observed in approximately 30% of systemic lupus erythematosus (SLE) patients, affecting various ocular structures. These ocular lesions, including lupus retinopathy and lupus-associated optic neuropathy, contribute to vision loss and serve as important indicators of SLE disease activity (1, 2). Early detection of preclinical retinal microvascular and microstructural alterations is crucial for preventing irreversible ocular and systemic complications and predicting prognosis (1, 3, 4).

Optical coherence tomography angiography (OCTA) is a non-invasive technology that enables high-resolution visualization of retinal vasculature, providing valuable insights into retinal vascular pathologies (5). Previous studies have shown that peripapillary retinal nerve fiber layer (RNFL) thickness and ganglion cell layer (GCL) thickness were vital parameters for glaucoma follow-up, reflecting prognosis and disease severity (6). Additionally, SLE patients exhibit thinning of the RNFL and GCL, suggesting neurodegeneration and early cognitive impairment (7).

OCTA has also revealed reductions in macular vessel density (VD) and foveal avascular zone (FAZ) area size in SLE patients without retinopathy, indicating microvasculature alterations even before ocular involvement. Furthermore, reduced retinal capillary vessel density has been observed in SLE patients with lupus nephritis (LN), suggesting potential early systemic vascular involvement (1, 8, 9). Recently, in our cross-sectional investigation (10), we utilized OCTA to image the microvasculature of the retina. Notable reductions in macular vessel density were observed, specifically within the superficial capillary plexus (SCP), among SLE patients without retinopathy compared to controls (10). These findings highlight the potential role of OCTA as a non-invasive tool for the early detection of systemic vascular changes and associated kidney impairment in lupus patients (3, 11).

It is well known that hydroxychloroquine (HCQ), a commonly used treatment for SLE, can lead to retinal toxicity by binding to melanin in retinal pigment epithelium (RPE) cells (12). This leads to disruptions in retinal cell metabolism and damage to the photoreceptors and outer nuclear layer of the retina (13, 14). Early detection of HCQ-induced retinopathy before RPE cell damage occurs is crucial for preserving vision, as the condition is irreversible and may lead to blindness.

As the number of biological treatment options continues to grow in the field of SLE therapy, there is a growing interest in investigating the effects of these medications on ocular health. Despite the growing body of research on ocular involvement in SLE, the effects of newer biological treatments, such as Belimumab, on ocular health remain largely unexplored.

Belimumab, a recombinant human IgG-1λ monoclonal antibody that inhibits B-cell activating factor, is approved for treating active autoantibody-positive SLE patients (15). It is part of the standard treatment for SLE and LN according to the European League Against Rheumatism (EULAR) 2023 guidelines (16). Recent research has demonstrated that Belimumab holds promise in the treatment of Graves’ Ophthalmopathy (GO) (17, 18). However, there is currently no research indicating its effects on the ocular vasculature of SLE patients.

The objective of this study is to conduct a detailed evaluation of ocular involvement in SLE and LN patients and investigate the influence of Belimumab on retinal microvascular density in these individuals.

## 2 Materials and methods

### 2.1 Patients

A total of 54 female patients with SLE from the Second Affiliated Hospital of Guangzhou Medical University were enrolled between September 2019 and October 2023. All SLE patients were diagnosed according to the 2019 EULAR/ACR classification criteria for SLE (19). 21 of the 54 patients included in the study met the diagnostic criteria for LN (20). SLE disease activity was evaluated utilizing the systemic lupus erythematosus disease activity index (SLEDAI)-2K score (21). In the study of Belimumab’s therapeutic effects, we included patients who had received Belimumab ≥8 times for the Belimumab treatment group, consistent with the dosing regimen used in the BLISS-LN trial (15). This cutoff corresponds to 24 weeks of treatment, which is generally sufficient to observe therapeutic effects.The ocular inclusion criteria were defined as follows: (1) best-corrected visual acuity better than 0.1 LogMAR; (2) intraocular pressure < 21 mmHg; (3) spherical equivalent < +2.5 D or > -6.0 D. Exclusion criteria encompassed SLE patients presenting with acute infections, malignancies, and other inflammatory diseases. We also excluded those treated with other biologic agents. The ocular exclusion criteria were defined as follows: (1) spherical equivalent > +6.0 D or <-6.0 D; (2) axial length ≥26 mm; (3) any ocular pathological changes detected on slit lamp, fundus color photography, or optical coherence tomography imaging; (4) history of previous ocular diseases such as glaucoma, cataract or ocular surgery, including refractive surgeries. In cases where both eyes fulfilled the inclusion criteria, data analysis was conducted on the right eye of each participant to prevent any potential bias that could result from the correlation between both eyes of the same individual. Informed consent was obtained from all participants, and the study received approval from the Ethics Committee of the Second Affiliated Hospital of Guangzhou Medical University.

### 2.2 Clinical and laboratory data and rheumatological treatment

Demographic data, clinical manifestations, and laboratory data were retrieved from the medical records of patients. Patient characteristics encompassed gender, age, disease duration, SLEDAI score, anti ds-DNA antibody positivity, C3, C4, erythrocyte sedimentation rate (ESR), C-reactive protein (CRP), albumin, creatinine, glomerular filtration rate (GFR), 24-hour proteinuria and anticardiolipin antibodies(ACL IgG, ACL IgA, ACL IgM, and anti-beta-2 glycoprotein I antibodies, Anti-β2GPI). We also collected data on the usage of Belimumab, HCQ, prednisolone, methotrexate(MTX), cyclophosphamide, mycophenolate mofetil(MMF), cyclophosphamid, cyclosporin A, FK506 in the included patients.

### 2.3 Ophthalmologic evaluation

**Figure 1:**
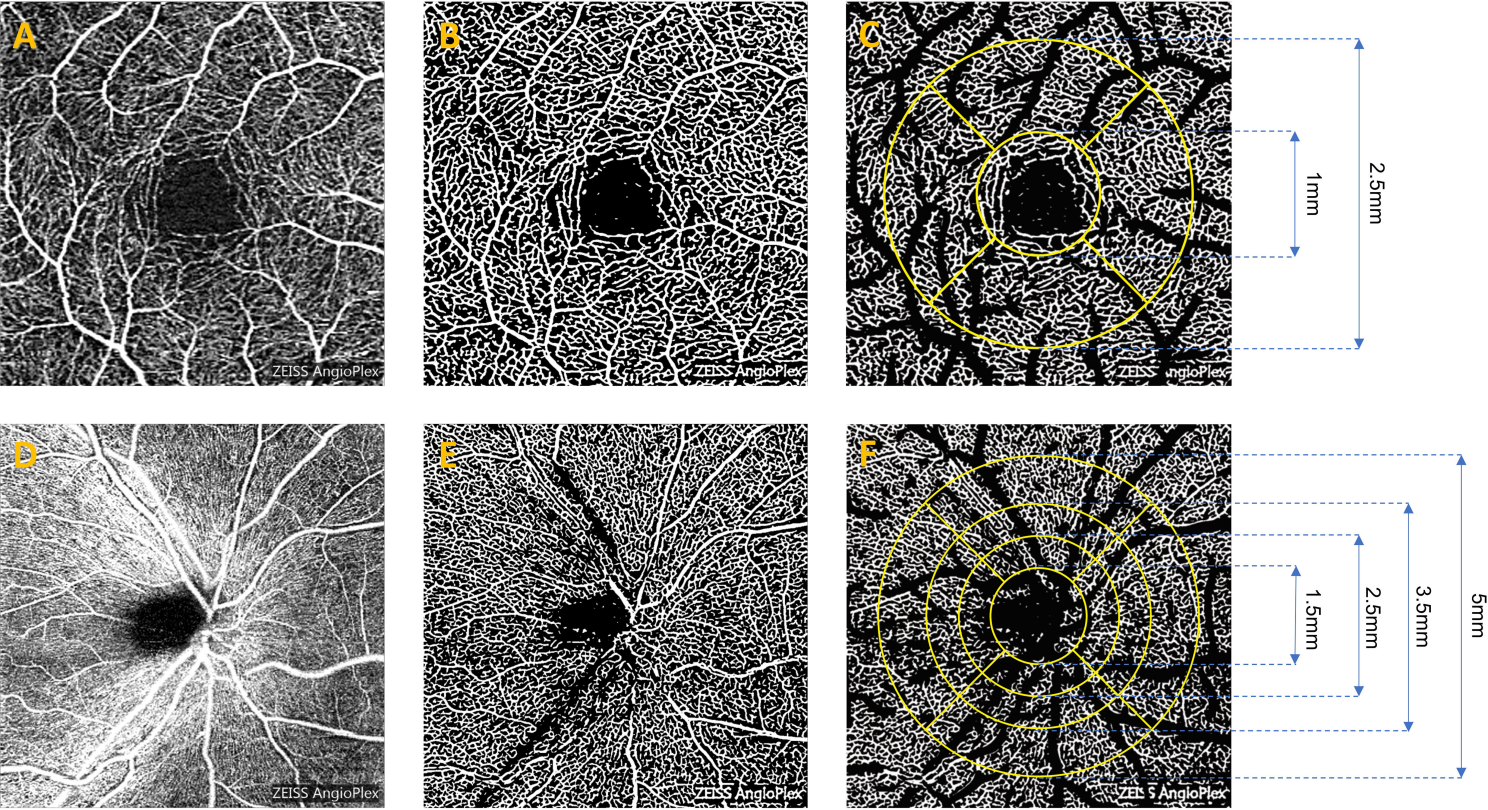
Representative OCTA images for measuring retinal microvasculature parameters in SCP. The original OCTA image revealed macular vessels **(A)** and peripapillary vessels **(D)**. The binarized image highlighted macular vessels **(B)** and peripapillary vessels **(E)**. After the removal of large vessels, the image displayed macular vessels **(C)** and peripapillary vessels **(F)**, which were then used to measure the corresponding subregions. OCTA, Optical Coherence Tomography Angiography; SCP, Superficial Capillary Plexus.

Every participant in the study received a comprehensive ophthalmological examination, which included best corrected visual acuity (BCVA), air puff intraocular pressure measurement (IOP), and OCTA examination. A standard LogMAR chart was used to evaluate BCVA in individual eyes for central visual acuity assessment following the Early Treatment of Diabetic Retinopathy Study (ETDRS) protocol (22). The evaluation incorporated the relevant macular and optic disc parameters. OCTA was used to measure RNFL thickness, GCL thickness, SCP-VD(%), and vessel length density (VLD)(%) (**Figure 1**). Similarly, the identical method was used to measure deep capillary plexus vessel density (DCP-VD) (%) and VLD (%). The FAZ area (mm^2^) and perimeter (mm) for both groups were also evaluated. Regarding the region of interest for analysis, the macular region was a circular region centered on the macula with a diameter of 1 mm and 2.5 mm respectively. The optic disc area was a circular region centered on the optic disc, with diameters of 1.5 mm, 2.5 mm, 3.5 mm, and 5 mm respectively. Parafoveal refers to the macular area 0.5–1.5 mm from the foveal center, and peripapillary denotes the optic disc region surrounding the optic nerve head. Macular VD was calculated using the ratio of flow pixels to total pixels. Macular VLD was calculated through the ratio of vessel length to total area. FAZ area and perimeter were calculated through manual delineation of the macular central avascular zone. The whole image corresponded to the complete 3 x 3 mm^2^ area centered on the macula or the entire 6 x 6 mm^2^ area centered on the optic disc. The analysis of OCTA images was performed using ImageJ software (https://imagej.net/Fiji; NIH, Bethesda, MD) following established quantification protocols (23, 24).

### 2.4 Statistical analysis

SPSS software (version 25.0), R Statistical Software (http://www.R-project.org, The R Foundation), and Free Statistics analysis platform were used for analysis. The normality of data was assessed using the Shapiro-Wilk test. Normally distributed continuous variables are presented as mean ± standard deviation and compared using the independent t-test. Non-normally distributed variables are presented as median (interquartile range) and compared using the Mann-Whitney U test. Categorical data are expressed as frequencies and analyzed using the chi-square test. For small samples, Fisher’s exact test was applied. Pearson’s correlation coefficient was used to assess linear relationships between normally distributed continuous variables, while Spearman’s rank correlation was used for non-normally distributed continuous and categorical variables. Multiple linear regression analysis was used to identify factors associated with reduced vascular density. No formal correction for multiple comparisons was applied due to the exploratory nature of the study and the potential risk of Type II errors. Statistical significance was defined as P < 0.05.

## 3 Results

### 3.1 Baseline characteristics of LN and non-LN patients

Twenty-one SLE patients with LN and 33 SLE patients without LN were recruited in this cross-sectional study. All the collected data regarding patients’ characteristics, SLE activity, and treatment are summarized in **Table 1**. No statistically significant differences were observed between the two groups in terms of age, gender, disease duration, SLEDAI score, anti ds-DNA antibody positivity, ESR, CRP, C3, C4, albumin, creatinine, GFR, and anticardiolipin antibodies (all P>0.05). LN patients exhibited a notable elevation in 24-hour urine protein levels compared to non-LN patients (961.0mg/d vs. 114.7mg/d, P<0.001). Additionally, there were no significant differences observed in visual acuity and IOP between the two groups of patients. We analyzed two groups of patients receiving rheumatological treatment. Our study results suggested that a higher proportion of patients with LN use prednisolone (90.5% vs 66.7%), MMF(76.1% vs 42.4%), and Belimumab (71.4% vs 36.4%) compared to patients with non-LN. (all P<0.05) (**Table 1**).

**Table 1:** Demographic and clinical characteristics in SLE patients with and without LN.

Variables	Non-LN patients (n = 33)	LN patients (n = 21)	P
Demographic characteristics			
Gender(Female)	33	21	
Age at study, year (mean ± s.d.)	36.8 ± 12.2	32.3 ± 7.7	0.139
Clinical features			
SLE duration, year	1.0 (0.3, 10.0)	3 (0.7, 7.5)	0.879
SLEDAI score	13.0(8.8, 16.5)	14.0 (11.0, 16.0)	0.891
Anti ds-DNA antibody positivity(%)	33 (100)	21 (100)	
Serological and urinary characteristics			
C3 complement (0.7-1.4), g/L	0.8 (0.6, 0.9)	0.7 (0.3, 1.0)	0.779
C4 complement (0.1-0.4), g/L	0.1 (0.1, 0.2)	0.1 (0.1, 0.2)	0.913
ESR (<40), mm/h	24.0 (13.2, 52.0)	60.0 (21.0, 73.0)	0.082
CRP (≤6), mg/L	3.4 (0.8, 10.4)	4.6 (1.1, 17.0)	0.440
Albumin (40-55), g/L	36.3 (33.0, 38.4)	32.1 (24.5, 38.8)	0.152
Creatinine (41-81), umol/L	57.5 (48.8, 63.2)	60.0 (55.0, 85.5)	0.071
GFR (>90), ml/min/1.73 m²	112.0 (100.1, 131.9)	103.7 (69.2, 116.7)	0.056
24hUP (<500), mg/d	114.7 (79.0, 203)	961.0 (224, 2743)	**<0.001**
ACL IgG (0-20), CU	6.6 (3.5, 12.7)	7.6 (5.0, 8.7)	0.856
ACL IgM (0-20), CU	2.1 (1.0, 3.4)	2.2 (1.8, 4.4)	0.327
ACL IgA (0-20), CU	4.2 (2.6, 6.9)	7.8 (2.3, 9.8)	0.685
Anti-β2GPI (0-20), CU	8.8 (4.4, 12.8)	9.1 (3.8, 13.3)	0.968
Ophthalmic conditions			
Visual acuity (logMAR)	-0.1 (-0.1, 0.0)	0.0 (-0.1, 0.0)	0.871
IOP (mmHg)	14.2 (12.9, 15.3)	15.0 (10.2, 15.5)	0.858
Rheumatological treatment ever, n (%)			
Prednisolone	22 (66.7)	19 (90.5)	**0.046**
Hydroxychloroquine	21 (63.6)	17 (81.0)	0.333
HCQ < 5years/ HCQ > 5 years	11(52.3)	8(47)	
Methotrexate	7 (21.2)	2 (9.5)	0.456
Cyclophosphamid (oral or IV)	NA	7 (33.3)	NA
Mycophenolate mofetil	14 (42.4)	16 (76.1)	**<0.001**
Cyclosporin A	5 (15.2)	1 (4.7)	0.386
FK506	NA	3 (14.3)	NA
Belimumab	12 (36.4)	15 (71.4)	**0.012**
Cumulative dose of immunosuppressants			
HCQ daily cumulative dose(mg/kg)	2.6 (0.0, 6.8)	4.9 (2.8, 6.9)	0.234
HCQ cumulative dose(g/kg)	0.7 (0.0, 12.6)	2.0 (0.1, 10.8)	0.659
GC cumulative dose(g/kg)	1.9 (0.0, 12.3)	4.2 (1.6, 19.9)	0.172

Values are median (interquartile range, IQR) unless stated otherwise; s.d., standard deviation; NA, not applicable; SLE, systemic lupus erythematosus; LN, lupus nephritis; non-LN, non-lupus renal disease; SLEDAI, systemic lupus erythematosus disease activity index; A positive result for anti-dsDNA antibodies indicates an antibody titer greater than 1:100. GFR, glomerular filtration rate; 24hUP, 24-hours urine protein; ACL, Anti-cardiolipin antibodies; anti-β2 GPI, Anti-β2-glycoprotein I antibodies; IOP, intraocular pressure; ‘Ever’ refer to use of immunosuppressants since the diagnosis of SLE; Receiving Belimumab treatment refers to the use of Belimumab more than 8 times. HCQ, Hydroxychloroquine; GC, Glucocorticoids; Normal value ranges are given in parentheses after the indicator. Bold values indicate statistically significant results (P < 0.05).

### 3.2 Retinal microvascular assessment by OCTA

#### 3.2.1 Retinal microvascular differences between LN and Non-LN patients

##### 3.2.1.1 Retinal nerve fiber layer thickness and ganglion cell layer thickness

Our findings did not show any significant difference in the thickness of the RNFL and GCL between the LN and non-LN groups. (all P>0.05, **Supplementary Table S1**).

##### 3.2.1.2 Superficial and deep capillary density in different areas of the macula

**Figure 2:**
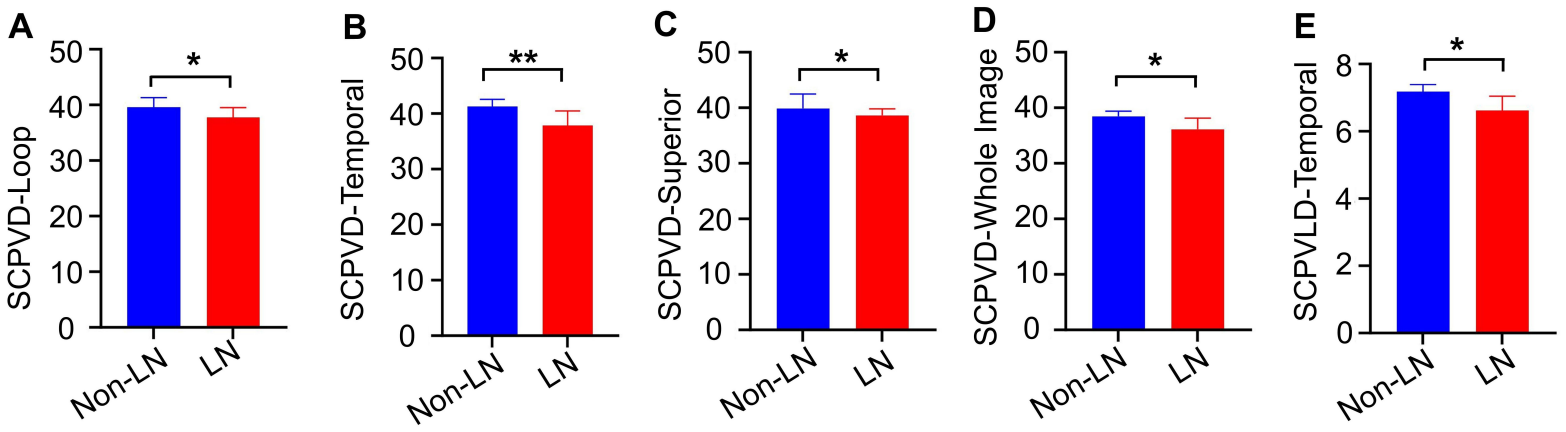
Comparative analysis of retinal microvascular density and vessel length density between LN patients and non-LN patients. We found that compared to non-LN patients, LN patients had lower vessel densities in superficial capillary plexus (SCP-VD)in the loop **(A)**, temporal **(B)**, superior **(C)**, and the whole image **(D)** areas, as well as lower superficial capillary plexus vessel length density (SCP-VLD) in the temporal area **(E)**. LN, lupus nephritis;*P<0.05, **P<0.01.

LN patients exhibited diminished superficial vasculature density (SCP-VD) in various regions, encompassing the loop, superior side, temporal side, and whole image region (all *P*<0.05). Additionally, a notable reduction in superficial vascular length density (SCP-VLD) was observed in the temporal side of LN patients (**Table 2**, **Figure 2**). 

**Table 2:** Comparison of macular and optic disc vascular density and vessel length density between SLE patients with and without LN.

Variables median (IQR)	Non-LN patients (n = 33)	LN patients (n = 21)	P
Macula			
SCP parafoveal VD(%)			
1mm Circle	21.6 (17.9, 23.4)	22.4 (18.3, 23.8)	0.380
2.5mm Circle	37.0 (34.7, 38.1)	35.1 (33.6, 36.4)	0.080
Loop	39.6 (37.6, 41.3)	37.7 (35.9, 39.4)	**0.046**
Superior	39.9 (37.7, 42.5)	38.7 (36.8, 39.4)	**0.028**
Nasal	40.0 (36.1, 41.7)	40.0 (37.7, 41.0)	0.908
Inferior	39.3 (37.5, 40.8)	37.6 (34.9, 39.3)	0.134
Temporal	41.3 (37.5, 42.6)	37.9 (35.5, 40.4)	**0.007**
Whole Image	38.5 (36.2, 39.1)	36.2 (35.4, 38.1)	**0.037**
SCP parafoveal VLD(%)			
1mm Circle	3.7 (3.0, 4.1)	4.0 (3.3, 4.1)	0.500
2.5mm Circle	6.4 (5.8, 6.7)	5.9 (5.8, 6.4)	0.108
Superior	6.9 (6.4, 7.3)	6.5 (6.1, 6.9)	0.059
Nasal	7.0 (6.1, 7.3)	6.9 (6.4, 7.2)	0.880
Inferior	6.8 (6.4, 7.1)	6.5 (6.0, 7.0)	0.092
Temporal	7.2 (6.7, 7.4)	6.6 (6.2, 7.0)	**0.014**
Whole Image	6.7 (6.2, 6.9)	6.3 (6.0, 6.7)	0.056
DCP parafoveal VD(%)			
1mm Circle	13.6 (11.3, 19.9)	17.2 (12.4, 19.7)	0.380
2.5mm Circle	36.8 (34.4, 38.5)	37.3 (35.3, 39.0)	0.325
Loop	41.1 (39.3, 42.2)	41.3 (39.5, 42.3)	0.389
Superior	42.0 (39.5, 43.7)	42.0 (39.0, 43.4)	0.915
Nasal	39.4 (37.4, 42.2)	40.8 (38.5, 44.5)	0.125
Inferior	40.3 (39.2, 41.8)	40.0 (38.6, 42.6)	0.352
Temporal	42.1 (39.7, 44.7)	41.0 (39.5, 42.4)	0.370
Whole Image	38.8 (36.8, 40.0)	39.4 (38.1, 40.3)	0.440
DCP parafoveal VLD(%)			
1mm Circle	2.8 (2.2, 3.9)	3.5 (2.5, 4.0)	0.283
2.5mm Circle	6.5 (6.2, 6.9)	6.7 (6.4, 7.0)	0.245
Superior	7.4 (6.9, 7.9)	7.4 (7.1, 7.8)	0.965
Nasal	7.1 (6.7, 7.3)	7.5 (6.9, 7.8)	0.101
Inferior	7.2 (6.9, 7.4)	7.2 (6.9, 7.7)	0.267
Temporal	7.4 (7.0, 8.0)	7.3 (7.1, 7.5)	0.356
Whole Image	7.0 (6.6, 7.2)	7.1 (6.8, 7.3)	0.308
FAZ			
FAZ-area (mm^2^)	0.4 (0.3, 0.5)	0.3 (0.3, 0.5)	0.325
FAZ-circle (mm)	3.8 (3.1, 4.6)	3.3 (3.0, 4.0)	0.361
Optic disc			
SCP peripapillary VD(%)			
1.5mm Circle	11.3 (8.2, 14.3)	14.7 (11.4, 16.6)	**0.022**
2.5mm Circle	21.3 (17.4, 23.6)	22.6 (21.2, 23.9)	0.112
3.5mm Circle	26.1 (23.7, 28.0)	27.6 (26.1, 29.0)	0.143
5.0mm Circle	28.4 (25.9, 30.3)	30.4 (27.9, 32.0)	0.164
Inner Circle			
Superior	26.2 (21.5, 28.4)	24.4 (22.9, 28.2)	0.965
Nasal	28.0 (20.3, 32.3)	28.3 (25.8, 32.2)	0.472
Inferior	25.3 (20.5, 27.9)	26.3 (22.3, 30.7)	0.104
Temporal	27.9 (22.6, 32.6)	34.3 (26.1, 37.1)	**0.046**
Middle Circle			
Superior	30.2 (28.2, 32.6)	31.3 (26.5, 34.4)	0.291
Nasal	31.3 (27.3, 35.8)	32.7 (28.5, 36.0)	0.908
Inferior	30.7 (27.8, 32.6)	31.4 (27.2, 32.7)	0.790
Temporal	34.4 (31.2, 36.9)	34.9 (31.8, 39.6)	0.409
Outer Circle			
Superior	31.8 (29.6, 34.4)	33.6 (27.3, 35.3)	0.540
Nasal	30.5 (27.8, 33.9)	31.9 (26.9, 34.8)	0.894
Inferior	31.4 (29.2, 33.2)	31.7 (29.6, 36.1)	0.316
Temporal	32.5 (29.7, 35.6)	33.5 (28.6, 37.3)	0.576
Whole Image	29.6 (27.5, 31.3)	29.9 (28.3, 32.9)	0.389
DCP peripapillary VD(%)			
1.5mm Circle	18.5 (12.5, 25.4)	17.2 (12.3, 28.1)	0.601
2.5mm Circle	20.0 (19.2, 21.2)	19.1 (17.6, 20.8)	0.316
3.5mm Circle	22.8 (21.4, 24.1)	23.3 (20.2, 24.6)	0.576
5.0mm Circle	24.5 (23.0, 25.9)	24.6 (22.1, 26.0)	0.979
Inner Circle			
Superior	21.9 (19.6, 24.9)	20.9 (16.7, 22.9)	0.129
Nasal	22.7 (19.6, 26.2)	22.7 (17.9, 29.1)	0.908
Inferior	22.1 (19.8, 25.9)	23.5 (19.8, 25.3)	0.783
Temporal	27.9 (23.9, 30.9)	26.5 (22.3, 27.6)	0.153
Middle Circle			
Superior	23.9 (21.9, 25.2)	25.4 (21.8, 26.7)	0.540
Nasal	28.4 (24.4, 32.2)	25.6 (22.7, 30.9)	0.389
Inferior	25.8 (22.7, 28.2)	24.2 (21.8, 26.3)	0.238
Temporal	27.6 (23.8, 29.7)	26.1 (24.6, 31.5)	0.763
Outer Circle			
Superior	25.2 (22.6, 27.4)	26.0 (23.9, 27.6)	0.472
Nasal	28.9 (26.0, 32.2)	31.4 (27.8, 34.5)	0.081
Inferior	25.5 (22.3, 27.2)	25.3 (22.6, 27.1)	0.922
Temporal	24.8 (22.2, 29.9)	24.7 (18.4, 31.0)	0.613
Whole Image	25.9 (23.5, 27.6)	25.2 (23.8, 28.5)	0.783

Values are median (interquartile range, IQR); SCP, superficial capillary plexus; DCP, deep capillary plexus; VD, vessel density; VLD, vessel length density; FAZ, foveal avascular zone; Parafoveal: macular region 0.5–1.5 mm from the foveal center; Peripapillary: optic disc region surrounding the optic nerve head. Bold values indicate statistically significant results (P < 0.05).

##### 3.2.1.3 Foveal avascular zone parameters

A comparative analysis of FAZ parameters was undertaken between patients with LN and those without LN. Nevertheless, no statistically significant differences were identified in either FAZ area or FAZ circle measurements between the two groups (all *P*>0.05, **Table 2**).

##### 3.2.1.4 Superficial and deep capillary density in different areas of the optic disc

Patients with LN exhibited elevated SCP-VD in the optic disc region, notably within the 1.5mm circle and temporal side of the inner circle (all *P*<0.05) (**Table 2**).

#### 3.2.2 Correlation of vascular parameters with clinical indicators

Our results showed that age, CRP, SLEDAI score and 24-hour urine protein were negatively correlated with VD on multiple sides (**Supplementary Table S2**).

#### 3.2.3 Retinal vascular changes associated with rheumatological treatments

##### 3.2.3.1 Belimumab

We investigated the vascular density of the superficial and deep capillary plexuses in SLE patients who either used(≥ 8 times) or did not use Belimumab. Notably, our analysis showed that patients who received Belimumab for more than 8 times showed a significant decrease in DCP-VD, particularly in the superior side of the inter circle(19.7 vs 23.0; P=0.023) (**Table 3**).

**Table 3:** Comparison of macular and optic disc vascular density and vessel length density among SLE patients using Belimumab.

Variables median (IQR)	Without Belimumab (n = 27)	With Belimumab (≥8times) (n = 27)	P
Macula			
SCP parafoveal VD (%)			
1mm Circle	22.1 (19.3, 23.5)	21.8 (17.5, 23.8)	0.952
2.5mm Circle	36.4 (34.6, 38.0)	35.8 (33.5, 37.9)	0.320
Loop	39.5 (37.5, 41.1)	38.4 (35.8, 40.2)	0.250
Superior	39.2 (37.5, 42.0)	38.7 (36.7, 40.3)	0.355
Nasal	40.7 (37.2, 42.4)	39.2 (36.7, 41.4)	0.164
Inferior	39.3 (35.9, 40.9)	38.5 (35.8, 39.8)	0.355
Temporal	40.5 (37.5, 42.2)	39.0 (35.9, 41.2)	0.229
Whole Image	38.1 (36.3, 39.1)	37.0 (35.3, 38.6)	0.130
SCP parafoveal VLD (%)			
1mm Circle	3.8 (3.3, 4.1)	3.8 (2.9, 4.2)	0.876
2.5mm Circle	6.4 (5.8, 6.6)	6.2 (5.7, 6.6)	0.382
Superior	6.9 (6.4, 7.3)	6.7 (6.1, 7.0)	0.431
Nasal	7.1 (6.2, 7.4)	6.9 (6.3, 7.2)	0.272
Inferior	6.8 (6.2, 7.1)	6.7 (6.2, 7.0)	0.562
Temporal	7.0 (6.6, 7.3)	6.7 (6.2, 7.3)	0.223
Whole Image	6.7 (6.2, 6.9)	6.4 (6.1, 6.7)	0.174
DCP parafoveal VD (%)			
1mm Circle	13.6 (11.7, 20.3)	15.8 (11.2, 19.4)	0.869
2.5mm Circle	37.3 (35.7, 38.8)	36.7 (34.8, 37.7)	0.243
Loop	41.9 (40.1, 42.9)	40.5 (39.3, 42.0)	0.117
Superior	42.2 (40.0, 43.7)	41.3 (39.0, 43.6)	0.478
Nasal	41.8 (38.7, 43.8)	39.4 (36.8, 40.8)	0.110
Inferior	40.6 (37.6, 42.2)	40.2 (39.5, 42.0)	0.829
Temporal	42.1 (40.0, 44.2)	41.0 (39.3, 43.3)	0.320
Whole Image	39.5 (37.7, 40.5)	38.6 (37.2, 39.6)	0.110
DCP parafoveal VLD (%)			
1mm Circle	2.7 (2.4, 3.9)	3.2 (2.3, 4.0)	0.802
2.5mm Circle	6.7 (6.4, 6.9)	6.6 (6.3, 6.9)	0.551
Superior	7.5 (7.0, 7.9)	7.2 (7.0, 7.8)	0.551
Nasal	7.2 (6.8, 7.6)	7.1 (6.7, 7.4)	0.272
Inferior	7.1 (6.8, 7.5)	7.2 (7.0, 7.5)	0.775
Temporal	7.4 (7.2, 7.8)	7.3 (6.9, 7.8)	0.328
Whole Image	7.1 (6.8, 7.2)	7.0 (6.8, 7.2)	0.346
FAZ			
FAZ-area (mm^2^)	0.4 (0.3, 0.5)	0.3 (0.3, 0.5)	0.373
FAZ-circle (mm)	3.8 (3.1, 4.7)	3.4 (3.0, 4.3)	0.320
Optic disc			
SCP peripapillary VD (%)			
1.5mm Circle	11.3 (8.5, 15.1)	12.7 (10.7, 16.4)	0.397
2.5mm Circle	21.8 (18.7, 23.4)	22.6 (19.4, 24.4)	0.382
3.5mm Circle	26.2 (24.2, 28.2)	27.2 (24.8, 29.0)	0.622
5.0mm Circle	29.6 (27.0, 31.3)	29.3 (26.3, 31.3)	0.842
Inter Circle			
Superior	24.9 (22.1, 28.7)	26.3 (21.8, 28.3)	0.966
Nasal	27.8 (21.8, 32.0)	29.3 (25.6, 32.2)	0.551
Inferior	25.3 (21.6, 28.4)	25.9 (19.6, 29.9)	0.789
Temporal	27.9 (21.7, 33.2)	32.4 (25.8, 35.9)	0.197
Middle Circle			
Superior	31.6 (28.4, 33.7)	29.7 (26.2, 33.2)	0.264
Nasal	32.0 (29.0, 36.2)	31.8 (26.6, 33.9)	0.382
Inferior	30.2 (26.3, 32.6)	31.4 (27.8, 33.2)	0.243
Temporal	33.9 (30.1, 36.2)	35.0 (33.0, 39.8)	0.110
Outer Circle			
Superior	33.0 (31.1, 35.3)	30.2 (26.7, 34.5)	0.082
Nasal	32.3 (29.3, 34.3)	29.7 (26.4, 34.1)	0.280
Inferior	31.4 (28.8, 34.6)	31.5 (29.4, 34.8)	0.815
Temporal	32.7 (29.1, 36.0)	32.5 (29.2, 35.8)	0.647
Whole Image	30.2 (27.5, 32.4)	29.6 (27.8, 31.2)	0.539
DCP peripapillary VD (%)			
1.5mm Circle	20.1 (12.6, 27.2)	15.2 (11.9, 25.9)	0.411
2.5mm Circle	19.9 (18.6, 20.7)	20.0 (17.5, 21.2)	0.924
3.5mm Circle	22.8 (20.6, 24.3)	23.3 (20.8, 24.1)	0.924
5.0mm Circle	24.5 (22.7, 25.9)	24.7 (22.3, 26.1)	0.869
Inter Circle			
Superior	23.0 (20.3, 25.5)	19.7 (18.2, 22.8)	**0.023**
Nasal	22.7 (18.9, 27.2)	22.7 (18.5, 26.5)	0.966
Inferior	22.5 (19.3, 25.6)	23.7 (19.8, 25.8)	0.697
Temporal	26.7 (23.9, 30.0)	27.1 (23.3, 30.5)	0.979
Middle Circle			
Superior	24.3 (22.4, 25.9)	24.0 (20.8, 26.5)	0.574
Nasal	28.4 (24.6, 32.7)	26.1 (22.3, 29.8)	0.139
Inferior	24.7 (20.6, 26.1)	26.0 (24.1, 29.3)	0.085
Temporal	26.1 (22.8, 28.9)	26.9 (24.7, 31.7)	0.110
Outer Circle			
Superior	25.6 (24.5, 28.2)	24.9 (22.4, 27.0)	0.250
Nasal	30.2 (27.2, 32.7)	29.6 (26.0, 32.9)	0.802
Inferior	25.2 (21.7, 27.5)	25.7 (23.3, 27.0)	0.164
Temporal	24.5 (21.9, 27.3)	26.3 (19.6, 30.3)	0.574
Whole Image	25.9 (23.5, 27.9)	25.2 (23.8, 28.5)	0.910

SCP, superficial capillary plexus; DCP, deep capillary plexus; VD, vessel density; VLD, vessel length density; FAZ, foveal avascular zone; Parafoveal: macular region 0.5–1.5 mm from the foveal center; Peripapillary: optic disc region surrounding the optic nerve head. Bold values indicate statistically significant results (P < 0.05).

##### 3.2.3.2 Hydroxychloroquine

We also evaluated vascular density in the superficial and deep capillary plexuses of SLE patients based on HCQ treatment. In the macular region, SLE patients using HCQ for over 5 years showed significantly lower DCP-VD, particularly in the 1mm circle (12.3 vs. 17.1; P=0.029), 2.5mm circle (35.6 vs. 37.4; P=0.019), loop (39.9 vs. 41.9; P=0.016), and whole image area (38.1 vs. 39.5; P=0.019). DCP-VLD was also reduced in these patients at the 1mm circle (2.5 vs. 3.5; P=0.036), 2.5mm circle (6.4 vs. 6.7; P=0.025), and whole image areas(6.8 vs. 7.1; P=0.028) (**Table 4**).

**Table 4:** Comparison of macular and optic disc vascular density and vessel length density based on HCQ treatment duration.

Variables median (IQR)	<5 Years (n = 35)	≥5 Years (n = 19)	P
Macula			
SCP parafoveal VD (%)			
1mm Circle	22.1 (19.0, 23.7)	21.6 (14.5, 23.5)	0.273
2.5mm Circle	36.2 (33.6, 38.0)	36.1 (34.2, 37.4)	0.765
Loop	38.8 (35.8, 41.1)	39.4 (37.6, 40.6)	0.949
Superior	39.0 (36.4, 42.3)	39.1 (37.8, 40.3)	0.949
Nasal	40.0 (36.3, 41.6)	40.1 (37.6, 41.5)	0.779
Inferior	39.3 (35.9, 40.6)	38.5 (35.5, 39.8)	0.544
Temporal	40.4 (36.4, 42.0)	39.8 (36.9, 42.0)	0.906
Whole imagine	37.6 (35.3, 39.1)	37.9 (36.0, 38.8)	0.892
SCP parafoveal VLD (%)			
1mm Circle	3.8 (3.3, 4.1)	3.7 (2.6, 4.0)	0.351
2.5mm Circle	6.3 (5.8, 6.7)	6.4 (5.8, 6.5)	0.779
Superior	6.7 (6.2, 7.3)	6.7 (6.4, 7.0)	0.807
Nasal	7.0 (6.2, 7.3)	6.9 (6.4, 7.3)	0.993
Inferior	6.8 (6.3, 7.1)	6.7 (6.1, 7.0)	0.562
Temporal	7.0 (6.5, 7.3)	7.1 (6.3, 7.4)	0.593
Whole imagine	6.6 (6.1, 6.9)	6.6 (6.3, 6.7)	0.964
DCP parafoveal VD (%)			
1mm Circle	17.1 (12.2, 20.5)	12.3 (9.3, 17.5)	**0.029**
2.5mm Circle	37.4 (36.1, 39.1)	35.6 (34.7, 37.2)	**0.019**
Loop	41.9 (40.1, 42.9)	39.9 (39.1, 41.2)	**0.016**
Superior	42.2 (39.8, 44.0)	41.3 (39.0, 42.6)	0.166
Nasal	40.8 (38.5, 44.0)	39.4 (37.3, 40.2)	0.160
Inferior	40.7 (39.4, 42.1)	39.6 (36.8, 40.9)	0.084
Temporal	41.8 (40.6, 44.2)	40.3 (38.5, 42.5)	0.109
Whole imagine	39.5 (38.4, 40.4)	38.1 (36.4, 38.8)	**0.019**
DCP parafoveal VLD (%)			
1mm Circle	3.5 (2.5, 4.1)	2.5 (2.0, 3.4)	**0.036**
2.5mm Circle	6.7 (6.5, 7.0)	6.4 (6.2, 6.7)	**0.025**
Superior	7.5 (7.1, 7.9)	7.2 (7.0, 7.6)	0.140
Nasal	7.3 (6.7, 7.6)	7.0 (6.9, 7.1)	0.208
Inferior	7.3 (7.1, 7.6)	7.1 (6.5, 7.3)	0.054
Temporal	7.4 (7.2, 7.8)	7.1 (6.9, 7.5)	0.090
Whole image	7.1 (6.9, 7.3)	6.8 (6.6, 7.1)	**0.028**
FAZ			
FAZ-area(mm^2^)	0.4 (0.3, 0.5)	0.3 (0.3, 0.5)	0.971
FAZ-circle(mm)	3.4 (2.9, 4.6)	3.3 (3.0, 4.2)	0.744
Optic disc			
SCP peripapillary VD (%)			
1.5mm Circle	12.5 (9.9, 15.1)	11.3 (8.9, 17.6)	0.849
2.5mm Circle	22.4 (19.0, 23.8)	21.4 (18.7, 24.1)	0.556
3.5mm Circle	27.5 (24.1, 28.9)	27.0 (24.9, 28.0)	0.544
5.0mm Circle	29.6 (26.8, 31.8)	29.2 (26.8, 30.5)	0.644
Inter Circle			
Superior	26.1 (21.8, 29.2)	25.9 (22.0, 27.1)	0.360
Nasal	29.7 (21.6, 32.4)	27.8 (24.4, 31.6)	0.906
Inferior	26.3 (21.0, 29.7)	22.3 (19.1, 26.9)	0.243
Temporal	29.7 (24.0, 36.7)	27.9 (22.7, 34.7)	0.474
Middle Circle			
Superior	31.6 (28.2, 34.2)	29.1 (27.3, 32.6)	0.235
Nasal	31.9 (27.1, 36.1)	31.8 (29.0, 35.2)	0.935
Inferior	31.3 (27.8, 32.8)	30.2 (26.9, 32.6)	0.328
Temporal	35.1 (32.7, 38.8)	34.4 (29.5, 35.4)	0.202
Outer Circle			
Superior	33.1 (29.6, 35.3)	31.3 (29.1, 34.1)	0.258
Nasal	29.7 (26.0, 33.3)	32.8 (30.2, 34.6)	0.080
Inferior	32.4 (29.4, 35.1)	31.4 (29.2, 33.3)	0.360
Temporal	32.5 (29.1, 36.7)	32.7 (29.0, 35.1)	0.697
Whole image	30.6 (28.1, 32.7)	29.2 (27.5, 30.1)	0.087
DCP peripapillary VD(%)			
1.5mm Circle	24.0 (13.0, 27.9)	14.8 (10.9, 18.5)	**0.033**
2.5mm Circle	19.9 (18.5, 21.2)	19.9 (18.6, 20.6)	0.724
3.5mm Circle	23.0 (20.9, 24.0)	22.8 (20.0, 24.8)	0.964
5.0mm Circle	24.6 (22.6, 26.0)	24.5 (22.8, 26.4)	0.949
Inter Circle			
Superior	20.9 (19.1, 23.2)	21.9 (19.3, 24.9)	0.520
Nasal	22.5 (18.8, 26.1)	25.0 (19.5, 30.4)	0.281
Inferior	23.5 (19.9, 25.9)	21.7 (18.4, 25.2)	0.281
Temporal	27.9 (24.2, 30.9)	25.4 (23.3, 27.6)	0.150
Middle Circle			
Superior	24.4 (21.4, 26.4)	23.9 (22.3, 25.0)	0.738
Nasal	28.0 (23.5, 31.3)	25.9 (22.9, 30.4)	0.878
Inferior	25.3 (22.3, 27.1)	24.4 (22.5, 27.8)	0.878
Temporal	26.5 (24.2, 29.7)	27.7 (24.4, 31.0)	0.431
Outer Circle			
Superior	25.7 (23.7, 28.0)	24.9 (22.5, 26.2)	0.273
Nasal	29.1 (26.1, 32.3)	30.3 (27.8, 34.0)	0.298
Inferior	25.6 (22.6, 27.5)	25.2 (22.0, 26.3)	0.463
Temporal	24.8 (22.2, 28.4)	24.7 (19.0, 31.1)	0.657
Whole image	25.4 (23.9, 28.0)	25.2 (23.3, 27.7)	0.644

LN, lupus nephritis; HCQ, hydroxychloroquine; SCP, superficial capillary plexus; DCP, deep capillary plexus; VD, vessel density; VLD, vessel length density; FAZ, foveal avascular zone; Parafoveal: macular region 0.5–1.5 mm from the foveal center; Peripapillary: optic disc region surrounding the optic nerve head. Bold values indicate statistically significant results (P < 0.05).

##### 3.2.3.3 Glucocorticoids

Compared to those who never used GC, patients with GC use showed reduced SCP-VD on the inferior side of the optic disc’s middle circle (30.1 vs. 32.5; P=0.042), (**Supplementary Table S3**).

##### 3.2.3.4 Mycophenolate mofetil

Our results showed that MMF users had lower SCP-VD and SCP-VLD on the macular temporal side (37.2 vs. 40.6; P=0.020 and 6.5 vs. 7.1; P=0.045) and reduced DCP-VD in the optic disc’s 2.5mm circle (18.9 vs. 20.0; P=0.041) and temporal inner circle (24.4 vs. 28.3; P=0.012), with increased DCP-VD on the nasal outer circle (32.0 vs. 28.7; P=0.013) (**Supplementary Table S4**).

#### 3.2.4 Association between cumulative drug exposure and vascular density

**Figure 3:**
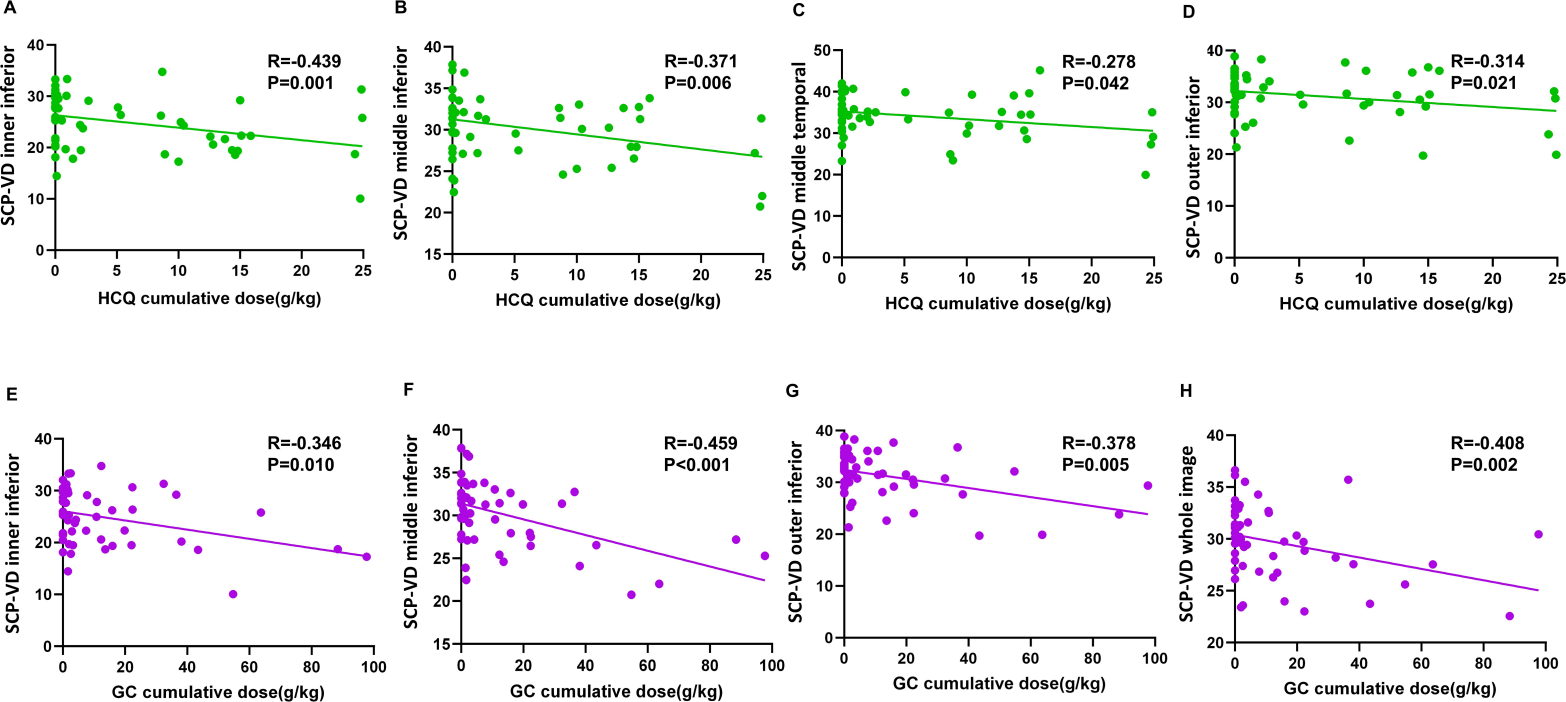
Linear regression scatter plots showed the correlation between retinal vascular density changes and cumulative doses of HCQ **(A–D)** or GC **(E–H)** in different retinal regions. HCQ, hydroxychloroquine; GC, glucocorticoid; SCP-VD, superficial capillary plexus vessel density.

The study explored the relationship between OCTA data and cumulative doses of HCQ and GC in SLE patients. The results indicate a negative correlation between cumulative HCQ doses and SCP-VD in multiple optic disc regions. Similarly, higher cumulative GC doses were negatively correlated with VD in various SCP and DCP regions of the optic disc area (all P<0.05, **Table 5**, **Figure 3**).

**Table 5:** Correlation of ocular indicators with cumulative doses of HCQ and GC in SLE patients.

Variables	HCQ cumulative dose		GC cumulative dose	
	R	P	R	P
Macula				
SCP parafoveal VD(%)				
1mm Circle	0.127	0.361	0.014	0.918
2.5mm Circle	0.096	0.491	-0.054	0.701
Loop	0.078	0.574	-0.037	0.790
Superior	-0.034	0.809	-0.162	0.241
Nasal	0.083	0.552	-0.132	0.341
Inferior	0.188	0.173	0.001	0.996
Temporal	-0.061	0.662	-0.061	0.661
Whole Image	0.108	0.437	-0.008	0.953
SCP parafoveal VLD(%)				
1mm Circle	0.16	0.247	0.051	0.715
2.5mm Circle	0.138	0.32	-0.002	0.990
Superior	0.007	0.96	-0.113	0.416
Nasal	0.078	0.575	-0.103	0.457
Inferior	0.214	0.12	0.03	0.831
Temporal	0.05	0.719	0.036	0.798
Whole Image	0.143	0.302	0.01	0.946
DCP parafoveal VD(%)				
1mm Circle	-0.017	0.903	0.045	0.745
2.5mm Circle	-0.01	0.943	-0.112	0.422
Loop	-0.016	0.907	-0.161	0.246
Superior	-0.027	0.846	-0.052	0.708
Nasal	0.01	0.945	-0.117	0.401
Inferior	0.128	0.355	0.018	0.896
Temporal	-0.102	0.461	-0.146	0.293
Whole Image	-0.088	0.526	-0.114	0.410
DCP parafoveal VLD(%)				
1mm Circle	0.008	0.953	0.069	0.620
2.5mm Circle	0.092	0.506	0.018	0.897
Superior	0.094	0.499	0.08	0.564
Nasal	0.094	0.497	-0.062	0.656
Inferior	0.169	0.223	0.068	0.627
Temporal	0.029	0.837	-0.052	0.708
Whole Image	0.037	0.79	0.003	0.981
FAZ				
FAZ-area (mm^2^)	0.016	0.909	0.035	0.804
FAZ-circle (mm)	0.098	0.48	0.091	0.514
Optic disc				
SCP peripapillary VD(%)				
1.5mm Circle	-0.067	0.631	-0.1	0.474
2.5mm Circle	-0.185	0.181	-0.212	0.124
3.5mm Circle	-0.251	0.067	-0.315	**0.020**
5.0mm Circle	-0.28	**0.041**	-0.354	**0.009**
Inner Circle				
Superior	-0.088	0.526	-0.002	0.986
Nasal	-0.026	0.853	-0.057	0.685
Inferior	-0.439	**0.001**	-0.346	**0.010**
Temporal	-0.133	0.336	-0.118	0.397
Middle Circle				
Superior	-0.137	0.325	-0.082	0.557
Nasal	-0.201	0.146	-0.259	0.059
Inferior	-0.371	**0.006**	-0.459	**<0.001**
Temporal	-0.278	**0.042**	-0.276	**0.043**
Outer Circle				
Superior	-0.27	**0.048**	-0.262	0.056
Nasal	-0.092	0.51	-0.159	0.252
Inferior	-0.314	**0.021**	-0.378	**0.005**
Temporal	-0.228	0.097	-0.155	0.264
Whole Image	-0.309	**0.023**	-0.408	**0.002**
DCP peripapillary VD(%)				
1.5mm Circle	0.234	0.089	-0.101	0.468
2.5mm Circle	-0.102	0.463	-0.238	0.083
3.5mm Circle	-0.173	0.21	-0.292	**0.032**
5.0mm Circle	-0.144	0.3	-0.262	0.056
Inner Circle				
Superior	-0.072	0.606	-0.096	0.488
Nasal	-0.103	0.46	-0.206	0.135
Inferior	-0.15	0.279	-0.299	**0.028**
Temporal	-0.247	0.072	-0.149	0.281
Middle Circle				
Superior	-0.053	0.703	-0.099	0.477
Nasal	-0.243	0.076	-0.289	**0.034**
Inferior	0.016	0.907	-0.165	0.232
Temporal	-0.035	0.803	0.005	0.971
Outer Circle				
Superior	-0.19	0.169	-0.239	0.082
Nasal	0.021	0.881	-0.004	0.979
Inferior	-0.145	0.294	-0.29	**0.033**
Temporal	-0.065	0.642	-0.102	0.464
Whole Image	-0.099	0.478	-0.286	**0.036**

HCQ, hydroxychloroquine; GC, glucocorticoids; SCP, superficial capillary plexus; DCP, deep capillary plexus; VD, vessel density; VLD, vessel length density; FAZ, foveal avascular zone; Bold values indicate statistically significant results (P < 0.05).

#### 3.2.5 Regression analysis of factors associated with parafoveal DCP vessel density

**Figure 4:**
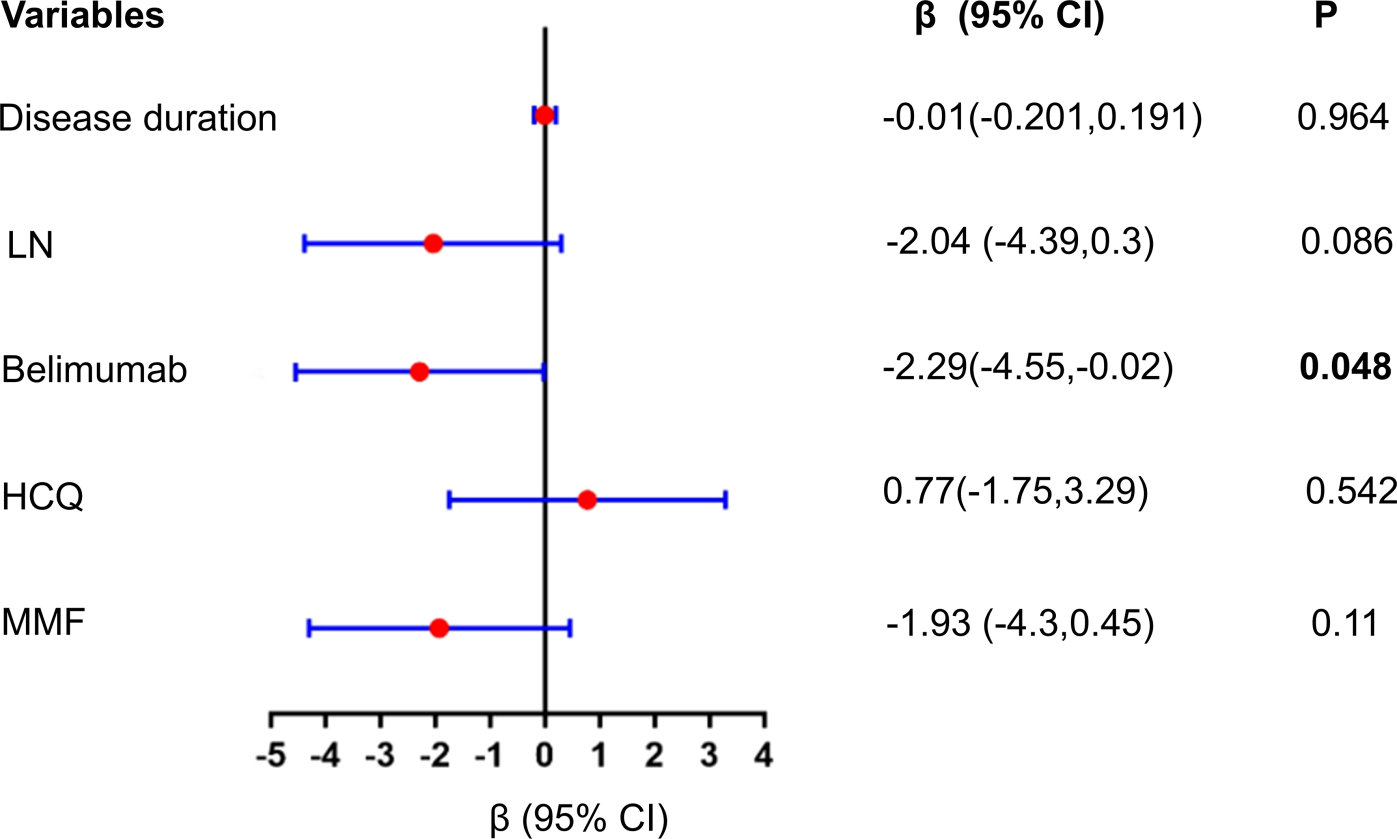
Univariate linear regression analysis of the impact of Belimumab and other factors on DCP peripapillary VD. Univariate regression analysis showed that LN, Belimumab treatment, and MMF treatment all had a negative impact on DCP peripapillary VD (β < 0). Notably, the effect of Belimumab treatment was statistically significant (P = 0.048); DCP, deep capillary plexus; VD, vessel density; LN, lupus nephritis; HCQ, Hydroxychloroquine; MMF, mycophenolate mofetil.

In our univariate regression analysis, it was found that LN, Belimumab treatment, and MMF treatment all had a negative impact on DCP-VD (β < 0). Notably, the effect of Belimumab treatment was statistically significant [β= -2.29 (-4.55~ -0.02), P = 0.048] (**Supplementary Table S5**, **Figure 4**). However, in the multivariate analysis, the effects of these agents were attenuated [β=-1.82(-4.17~0.53), P = 0.135] (**Supplementary Table S5**).

### 3.3 Comparison of cytokines and lymphocyte subsets before and after Belimumab treatment

**Figure 5:**
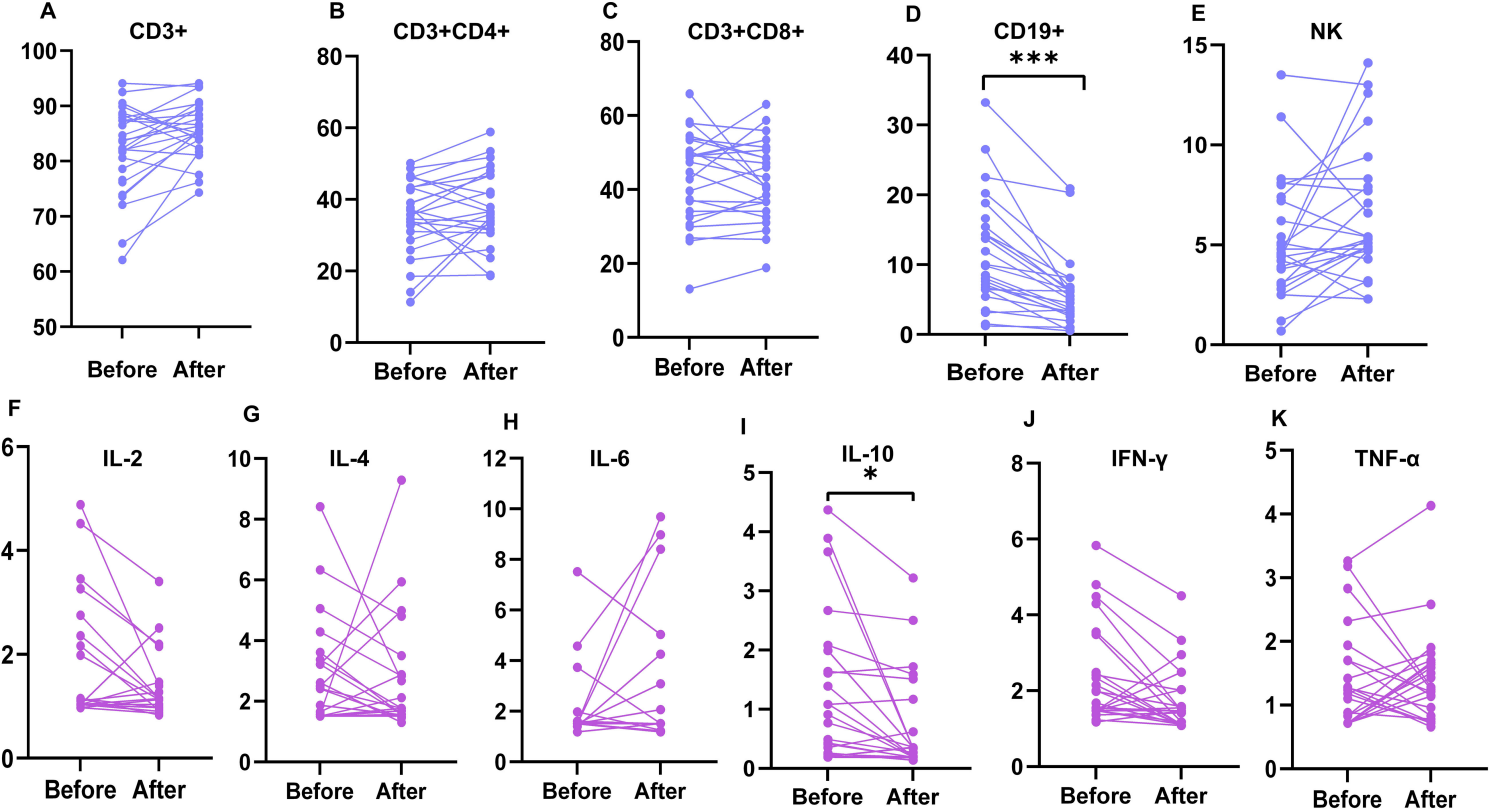
Comparison of lymphocyte subsets and cytokines between patients before and after treatment with Belimumab. The changes in lymphocyte subsets **(A–E)** and cytokines **(F–K)** in patients before and after treatment with Belimumab were compared. CD19+ B cells significantly decreased (***P < 0.001), and IL-10 levels increased (*P < 0.05) in treated patients. IL, interleukin; IFN-γ, interferon-gamma; TNF-α, tumor necrosis factor-alpha; NK, natural killer cells.

We found that patients treated with Belimumab showed a significant decrease in CD19+ B cells (5.2% vs 9.8% ; P < 0.001) and IL-10 levels (0.3 pg/ml vs 0.8 pg/ml ; P = 0.026). There were no statistically significant differences in changes in other lymphocyte subsets and cytokines before and after treatment (**Table 6**, **Figure 5**).

**Table 6:** Comparison of cytokines and lymphocyte subsets between patients before and after treatment with Belimumab.

Variables	Before Belimumab (n = 27)	After Belimumab (n=27)	P
Lymphocyte subsets			
CD3+(65-79),%	83.6 (77.6, 88.2)	85.3 (83.9, 89.2)	0.152
CD3+CD4+(34-52),%	35.7 (31.9, 43.2)	36.1 (31.5, 46.6)	0.563
CD3+CD8+(21-39),%	47.4 (33.4, 51.9)	42.1 (36.5, 50.2)	0.715
CD19+(9.02-14.1),%	9.8 (6.6, 14.9)	5.2 (3.3, 6.5)	**< 0.001**
NK(10.04-19.78),%	4.6 (3.5, 7.3)	5.3 (4.7, 8.2)	0.091
Cytokines			
IL-2 (≤4.34), pg/ml	1.0 (1.0, 2.3)	1.1 (1.1, 1.4)	0.764
IL-4 (≤2.90), pg/ml	2.4 (1.5, 3.5)	1.8 (1.6, 2.8)	0.991
IL-6 (≤5.04), pg/ml	1.5 (1.5, 1.6)	1.5 (1.5, 2.8)	0.246
IL-10 (≤5.00), pg/ml	0.8 (0.2, 2.4)	0.3 (0.2, 0.4)	**0.026**
IFN-γ (≤3.87), pg/ml	1.5 (1.5, 2.1)	1.5 (1.1, 1.9)	0.082
TNF-α (≤4.41), pg/ml	1.2 (0.7, 2.1)	1.2 (1.0, 1.6)	0.802

NK, natural killer cells; IL, Interleukin; IFN-γ, Interferon-gamma; TNF-α, tumor necrosis factor alpha. Normal value ranges are given in parentheses after the indicator. Bold values indicate statistically significant results (P < 0.05).

## 4 Discussion

SLE patients with LN experience higher morbidity and mortality rates compared to those without nephritis, leading to worse survival outcomes. Furthermore, lupus retinopathy can occur due to a vasculitic process affecting the microvasculature of the retina.The relationship between SLE and ocular vascular involvement is of particular clinical significance and requires comprehensive understanding. Previous studies found reduced retinal microvascular density in SLE patients, particularly those with renal involvement, compred to healthy controls (1, 3, 25). Recognizing the significance of ocular involvement is crucial for comprehensive management and improved outcomes in individuals with LN.

With the advent of steroids and immunosuppressive therapies, the incidence of retinal involvement in SLE patients ranges from 7-29%, which is associated with visual loss (26, 27). The main mechanisms leading to SLE retinopathy are the immune complex-mediated microangiopathic vasculopathy, secondary hypertension resulting in kidney involvement with activation of the renin-angiotensin-aldosterone system (RAAS), and the micro-thrombosis associated with either underlying endothelial injury or antiphospholipid (aPL) antibodies presence (28–30).In SLE patients, due to abnormal activity of the immune system, immune complexes may deposit in ocular tissues, which can lead to inflammation and damage to the retinal blood vessels, resulting in a decrease in retinal vascular density (28).

In SLE patients with early retinal lesions, the subjective perception of visual loss may be subtle due to the absence of prominent clinical manifestations. However, delayed recognition of visual impairment often coincides with the progression of retinal lesions, in concurrence with the advancing SLE pathology, rendering subsequent treatment interventions considerably more complex. Therefore, early detection and diagnosis of ocular involvement are crucial in SLE patients.

In recent years, there has been growing interest in the use of OCTA to detect early ocular vascular damage in systemic conditions, including arterial hypertension (31), diabetes (32), and chronic kidney disease (4). As a multisystemic disease, OCTA plays a particularly significant role in the early assessment of ocular vasculature in patients with SLE.

Wang et al. observed significant reductions in central macular thickness and in both the SCP-VD and DCP-VD of SLE patients compared to healthy controls as determined by OCTA. Additionally, no significant difference was found in retinal vascular densities between the LN and non-LN groups (33). Our research findings demonstrated that in LN patients, the loop area, superior, temporal, and whole image areas in the superficial capillaries exhibited significantly lower densities compared to the non-LN group (p <0.05), consistent with the findings of Conigliaro et al. Remarkably, we observed a significant negative correlation between vascular density and 24-hour urinary protein levels, further elucidating the association between ocular vascular density and renal impairment in patients. These patterns may reflect the cumulative burden of systemic inflammation and immunomodulation on the retinal microcirculation, supporting the utility of OCTA in capturing subclinical vascular damage.

The distinct retinal microvascular alterations in LN patients are strongly associated with shared pathophysiological mechanisms involving both ocular and renal microvasculature. Immune complex deposition induces endothelial activation and inflammation, leading to vascular damage (34). Elevated cytokines such as IL-6 and TNF-α exacerbate systemic inflammation, promoting endothelial dysfunction and microvascular remodeling (35).Furthermore, antiphospholipid antibodies contribute to thrombotic microangiopathy, resulting in vascular occlusion and ischemia (36).

Based on our findings of significantly reduced SCP-VD in LN patients, we further analyzed the associations between clinical parameters and vascular density. Taking into account the observed associations between age and CRP level changes with SCP-VD, therefore, we have considered that the reduction of SCP-VD is associated with vasculitis.

The retinal vasculature is one of the vessels that can be directly observed in SLE patients, and its performance often reflects the degree of systemic vascular damage. Similar to Conigliaro et al. and Ermurat et al. results, our study has revealed a negative correlation between the SLEDAI-2K scores and the retinal microvascular VD (1, 37).

While previous studies have reported enlargement in FAZ parameters in patients with SLE (38, 39), some other studies have failed to confirm this and reported no significant differences between SLE patients and control groups (40, 41). In our study, we didn’t observe an enlargement in the FAZ perimeter and FAZ area in LN patients. Based on our findings, we propose that the changes in vascular density in SLE are more prominent in the perifoveal area, while the parafovea and fovea are comparatively less affected.

In addition to the direct effects of SLE, drugs used in SLE therapy can potentially lead to retinal and visual impairment (42). HCQ was particularly associated with maculopathy and irreversible retinal damage (43). MTX can cause ischemic retinal complications, while cyclosporine can result in decreased visual acuity (44, 45).

To the best of our knowledge, this is the first investigation into the impact of Belimumab on retinal microvascular density in SLE patients. Our findings suggested that the use of Belimumab correlates with a decrease in vascular density.

Belimumab, as an emerging targeted therapy, is widely used in rheumatic diseases such as SLE, LN, and Sjögren’s syndrome, and has shown promising therapeutic outcomes (46–48). Therefore, it is crucial to closely monitor ocular involvement associated with the use of Belimumab to prevent severe visual impairment.

Notably, our research findings indicate a decrease in retinal microvascular vessel density in the patients treated with Belimumab, which has sparked great interest in us. Previous studies have shown promising efficacy of Belimumab in anti-neutrophil cytoplasmic antibody (ANCA)-associated vasculitis and cryoglobulinemic vasculitis (49, 50). However, our research results reveal a reduction in retinal microvascular vessel density in patients treated with Belimumab.

To investigate how Belimumab affects ocular vascular density, we compared cytokine and lymphocyte subset levels among patients before and after Belimumab treatment. We found that patients treated with Belimumab showed a significant decrease in CD19+ B cells and IL-10 levels. Previous studies showed that B-cell activating factor (BAFF) enhances angiogenesis and endothelial cell proliferation by upregulating vascular endothelial growth factor (VEGF) expression (51, 52). Furthermore, studies have indicated a correlation between CD19+ B cell numbers and VEGF expression levels. These cells are pivotal within the tumor microenvironment, influencing both VEGF expression and angiogenesis (53, 54). We hypothesize that the long-term use of Belimumab inhibits BAFF, subsequently altering B cell function and leading to a reduction in CD19+ cell counts, which ultimately affects VEGF expression and vascular density. Although prior immunological studies support this mechanistic pathway, it remains hypothetical due to the absence of direct VEGF measurements in our study. Additionally, our correlation regression analysis indicates that the use of Belimumab treatment negatively impacts DCP peripapillary VD. Our regression analysis, which adjusted for key confounders such as LN prevalence and disease duration (**Supplementary Table S6**), showed that while these adjustments attenuated Belimumab’s effects, the consistent negative trend observed across both univariate (β = -2.29, P = 0.048) and multivariate analyses (β = -1.82, P = 0.135) suggests a potential role for Belimumab in influencing retinal vascular density. This may justify the inclusion of routine OCTA screening in clinical follow-up protocols for patients with SLE, particularly those receiving biologic agents. However, due to the limited sample size in our study, expanding the cohort is essential to validate the reliability of our findings.

Previous studies have evaluated the effect of HCQ on retinal microvascular structure and suggested its potential protective effect. However, these studies did not conduct subgroup analyses considering factors like cumulative dose and duration of use (1, 55). In contrast, Mihailovic et al. performed a study with low- and high-risk subgroups based on HCQ use duration and cumulative dose, revealing that the protective effect was only present in the low-risk group (38). Our findings indicate that patients treated with HCQ for more than 5 years showed reduced DCP-VD in the 1.5 mm circle, 2.5mm circle and loop area. These results are consistent with the result of Jelena et al (14). We hypothesize that these differences may be attributed to prolonged disease duration and long-term HCQ use. It is worth noting that we conducted further analysis and found a negative correlation between retinal vascular density and the cumulative dose of HCQ and GC in patients. Higher cumulative doses of HCQ and GC may contribute to a reduction in retinal vascular density, emphasizing the potential influence of long-term HCQ treatment on retinal vascular density in SLE patients. Additionally, longer disease duration may also negatively impact retinal vascular density. These findings underscore the importance of regular monitoring and assessment of ocular health in SLE patients, especially those receiving long-term HCQ treatment.

Our study also has several limitations. Our cross-sectional study design limits the ability to establish causal relationships between retinal vascular density changes and Belimumab treatment. While we adjusted for confounders such as LN status and disease duration, the multifactorial nature of retinal microvascular changes makes it challenging to disentangle the effects of treatment from those of disease severity. Additionally, the absence of a healthy control group for comparison limits the generalizability of our findings. The observed associations, while statistically significant, require further investigation to confirm their clinical relevance. To address the limitations of this study, we are conducting ongoing longitudinal cohort studies incorporating serial OCTA imaging to investigate the cumulative effects of Belimumab on microvascular health, aiming to validate these findings and establish their clinical relevance.

## 5 Conclusion

In conclusion, our study found lower parafoveal SCP-VD of SLE-LN patients without retinopathy compared to SLE patients. Notably, Belimumab treatment resulted in a reduction in peripapillary DCP-VD area. These findings enhance our understanding of treatment effects on retinal vasculature in SLE. Our results highlight the potential of OCTA for early detection of retinal vascular damage in SLE-LN patients without retinopathy. Future studies with larger cohorts can provide further insights into these observations.

These findings support the value of incorporating routine OCTA screening into clinical follow-up for SLE patients, especially those undergoing long-term biologic therapy, to detect subclinical retinal changes. Such integration may facilitate earlier therapeutic intervention and more individualized disease management.

## Data Availability

The original contributions presented in the study are included in the article/**Supplementary Material**. Further inquiries can be directed to the corresponding authors.
