# Supplementary material for: Belimumab-driven reductions in retinal microvascular density assessed by optical coherence tomography angiography: insights from systemic lupus erythematosus patients
Source: Front Immunol. 2025 Jun 16;16:1511133. doi: 10.3389/fimmu.2025.1511133 (PMC12206790; doi:10.3389/fimmu.2025.1511133)
Supplement: Supplementary file 1 [file Table1.docx]

**Supplementary Table S1.Comparison of retinal nerve fiber layer (RNFL) thickness and ganglion cell layer (GCL) thickness between non-LN SLE patients and LN patients**

| **Variables**  **Median (IQR)** | **Non-LN**  **(n = 33)** | **LN**  **(n = 21)** | **P** |
| --- | --- | --- | --- |
| **RNFL(um)** |  |  |  |
| Temporal | 78.0 (69.0, 86.0) | 78.0 (66.2, 92.8) | 0.654 |
| Superior | 126.0(113.0,138.0) | 125.5(118.8,131.5) | 0.927 |
| Nasal | 64.0 (58.0, 71.0) | 68.5 (61.8, 74.0) | 0.082 |
| Inferior | 121.0(109.0,138.0) | 131.0(121.0,135.0) | 0.203 |
| Average | 97.0 (91.0, 103.2) | 100.2 (96.5, 103.6) | 0.354 |
| **GCL(um)** |  |  |  |
| Superior Temporal | 80.0 (78.0, 83.0) | 80.0 (78.0, 83.0) | 0.749 |
| Superior | 83.0 (79.2, 86.0) | 84.0 (81.5, 86.5) | 0.614 |
| Superior Nasal | 85.5 (82.0, 88.0) | 86.0 (83.0, 88.5) | 0.578 |
| Inferior Nasal | 83.0 (78.0, 85.8) | 83.0 (80.5, 87.0) | 0.465 |
| Inferior | 79.5 (77.0, 82.0) | 80.0 (77.5, 83.5) | 0.651 |
| Inferior Temporal | 81.0 (77.2, 82.8) | 82.0 (79.0, 85.5) | 0.523 |
| Average | 81.0 (79.0, 84.0) | 82.0 (80.0, 85.5) | 0.551 |

Notes: RNFL: retinal nerve fiber layer; GCL: ganglion cell layer; RNFL thickness was measured using quadrant partitions, while GCL thickness was measured using elliptic partitions. P values below 0.05 indicate statistical significance.

**Supplementary Table S2**. **Correlation of retinal vascular density with clinical variables in patients with systemic lupus erythematosus**

| **Variables** | **Age** | | **Disease**  **duration** | | | **SLEDAI**  **score** | | | **ESR** | | | **CRP** | | | **GFR** | | | **24-hour**  **urine protein** | | **Creatinine** | | |  |
| --- | --- | --- | --- | --- | --- | --- | --- | --- | --- | --- | --- | --- | --- | --- | --- | --- | --- | --- | --- | --- | --- | --- | --- |
|  | **R** | **P** | **R** | **P** | **R** | | **P** | **R** | | **P** | **R** | | **P** | **R** | | **P** | **R** | | **P** | **R** | **P** |  |  |
| **Macula** | | | | | | | | | | | | | | | | | | | | | | | |
| **SCP parafoveal VD(%)** | | | | | | | | | | | | | | | | | | | | | | | |
| 1mm Circle | -0.465 | **<0.001** | -0.223 | 0.111 | 0.054 | | 0.699 | -0.198 | | 0.156 | -0.280 | | **0.047** | -0.174 | | 0.222 | 0.096 | | 0.530 | 0.270 | 0.056 |  |  |
| 2.5mm Circle | -0.422 | **0.001** | -0.089 | 0.530 | -0.060 | | 0.672 | -0.232 | | 0.095 | -0.336 | | **0.016** | -0.091 | | 0.526 | -0.148 | | 0.331 | 0.162 | 0.256 |  |  |
| Loop | -0.349 | **0.010** | -0.011 | 0.937 | -0.106 | | 0.451 | -0.192 | | 0.168 | -0.315 | | **0.024** | -0.052 | | 0.716 | -0.207 | | 0.171 | 0.104 | 0.468 |  |  |
| Superior | -0.238 | 0.083 | 0.019 | 0.892 | -0.137 | | 0.327 | -0.179 | | 0.199 | -0.207 | | 0.145 | -0.004 | | 0.978 | -0.281 | | 0.061 | 0.047 | 0.742 |  |  |
| Nasal | -0.352 | **0.009** | -0.107 | 0.449 | 0.058 | | 0.683 | -0.036 | | 0.800 | -0.200 | | 0.160 | 0.007 | | 0.961 | 0.259 | | 0.086 | 0.132 | 0.356 |  |  |
| Inferior | -0.255 | 0.062 | -0.084 | 0.552 | -0.138 | | 0.324 | -0.176 | | 0.209 | -0.331 | | **0.018** | 0.017 | | 0.904 | -0.278 | | 0.065 | 0.055 | 0.701 |  |  |
| Temporal | -0.113 | 0.414 | 0.067 | 0.636 | -0.186 | | 0.182 | -0.166 | | 0.234 | -0.198 | | 0.165 | -0.099 | | 0.488 | -0.356 | | **0.016** | 0.093 | 0.514 |  |  |
| Whole Image | -0.391 | **0.003** | 0.022 | 0.875 | -0.041 | | 0.769 | -0.196 | | 0.159 | -0.278 | | **0.049** | -0.046 | | 0.751 | -0.182 | | 0.232 | 0.101 | 0.480 |  |  |
| **SCP parafoveal VLD(%)** | | | | | | | | | | | | | | | | | | | | | | | |
| 1mm Circle | -0.473 | **<0.001** | -0.211 | 0.134 | 0.041 | | 0.773 | -0.191 | | 0.170 | -0.289 | | **0.039** | -0.123 | | 0.390 | 0.068 | | 0.659 | 0.275 | 0.050 |  |  |
| 2.5mm Circle | -0.389 | **0.004** | -0.089 | 0.531 | -0.119 | | 0.398 | -0.199 | | 0.153 | -0.404 | | **0.003** | -0.147 | | 0.304 | -0.152 | | 0.320 | 0.238 | 0.093 |  |  |
| Superior | -0.205 | 0.138 | 0.015 | 0.915 | -0.151 | | 0.281 | -0.161 | | 0.250 | -0.237 | | 0.094 | -0.035 | | 0.806 | -0.294 | | 0.050 | 0.082 | 0.569 |  |  |
| Nasal | -0.348 | **0.010** | -0.154 | 0.275 | -0.039 | | 0.782 | -0.011 | | 0.936 | -0.292 | | **0.037** | -0.079 | | 0.583 | 0.188 | | 0.216 | 0.215 | 0.130 |  |  |
| Inferior | -0.212 | 0.124 | -0.091 | 0.519 | -0.151 | | 0.282 | -0.202 | | 0.146 | -0.374 | | **0.007** | -0.067 | | 0.639 | -0.330 | | **0.027** | 0.113 | 0.428 |  |  |
| Temporal | -0.101 | 0.469 | 0.079 | 0.577 | -0.230 | | 0.097 | -0.146 | | 0.296 | -0.242 | | 0.087 | -0.205 | | 0.150 | -0.315 | | **0.035** | 0.209 | 0.141 |  |  |
| Whole Image | -0.341 | **0.012** | 0.018 | 0.902 | -0.080 | | 0.568 | -0.200 | | 0.151 | -0.317 | | **0.024** | -0.150 | | 0.293 | -0.186 | | 0.221 | 0.191 | 0.179 |  |  |
| **DCP parafoveal VD(%)** | | | | | | | | | | | | | | | | | | | | | | | |
| 1mm Circle | -0.094 | 0.500 | -0.157 | 0.266 | 0.002 | | 0.987 | 0.014 | | 0.923 | -0.026 | | 0.857 | 0.073 | | 0.612 | 0.057 | | 0.709 | 0.082 | 0.569 |  |  |
| 2.5mm Circle | -0.297 | **0.029** | -0.312 | **0.024** | -0.004 | | 0.979 | 0.101 | | 0.473 | -0.138 | | 0.334 | -0.031 | | 0.828 | 0.136 | | 0.375 | 0.293 | **0.037** |  |  |
| Loop | -0.339 | **0.012** | -0.328 | **0.018** | -0.011 | | 0.937 | 0.094 | | 0.501 | -0.222 | | 0.117 | -0.100 | | 0.487 | 0.176 | | 0.247 | 0.362 | **0.009** |  |  |
| Superior | -0.284 | **0.037** | -0.153 | 0.278 | 0.078 | | 0.581 | -0.035 | | 0.806 | -0.064 | | 0.653 | -0.068 | | 0.636 | -0.047 | | 0.762 | 0.188 | 0.187 |  |  |
| Nasal | -0.112 | 0.418 | -0.207 | 0.141 | -0.096 | | 0.493 | 0.180 | | 0.198 | -0.054 | | 0.704 | -0.090 | | 0.529 | 0.257 | | 0.089 | 0.275 | 0.050 |  |  |
| Inferior | -0.200 | 0.147 | -0.247 | 0.077 | -0.023 | | 0.868 | 0.073 | | 0.602 | -0.316 | | **0.024** | -0.048 | | 0.740 | 0.085 | | 0.578 | 0.208 | 0.143 |  |  |
| Temporal | -0.171 | 0.217 | -0.181 | 0.200 | -0.015 | | 0.916 | 0.076 | | 0.587 | -0.109 | | 0.447 | -0.068 | | 0.633 | -0.075 | | 0.623 | 0.193 | 0.176 |  |  |
| Whole Image | -0.240 | 0.080 | -0.197 | 0.162 | 0.011 | | 0.939 | 0.176 | | 0.208 | -0.144 | | 0.315 | -0.021 | | 0.885 | 0.112 | | 0.464 | 0.235 | 0.097 |  |  |
| **DCP parafoveal VLD(%)** | | | | | | | | | | | | | | | | | | | | | | | |
| 1mm Circle | -0.046 | 0.743 | -0.166 | 0.240 | 0.022 | | 0.873 | -0.004 | | 0.977 | -0.004 | | 0.979 | 0.088 | | 0.540 | 0.075 | | 0.626 | 0.058 | 0.685 |  |  |
| 2.5mm Circle | -0.239 | 0.081 | -0.272 | 0.051 | -0.008 | | 0.952 | 0.025 | | 0.860 | -0.169 | | 0.237 | -0.074 | | 0.604 | 0.171 | | 0.261 | 0.326 | **0.020** |  |  |
| Superior | -0.269 | **0.049** | -0.131 | 0.356 | 0.058 | | 0.678 | -0.088 | | 0.533 | -0.108 | | 0.452 | -0.114 | | 0.426 | -0.003 | | 0.985 | 0.290 | **0.039** |  |  |
| Nasal | -0.121 | 0.383 | -0.244 | 0.081 | -0.100 | | 0.476 | 0.131 | | 0.349 | -0.071 | | 0.623 | -0.105 | | 0.463 | 0.255 | | 0.090 | 0.333 | **0.017** |  |  |
| Inferior | -0.224 | 0.104 | -0.253 | 0.071 | -0.016 | | 0.907 | 0.016 | | 0.908 | -0.396 | | **0.004** | -0.136 | | 0.340 | 0.159 | | 0.298 | 0.318 | **0.023** |  |  |
| Temporal | -0.175 | 0.206 | -0.184 | 0.192 | -0.069 | | 0.624 | 0.002 | | 0.986 | -0.164 | | 0.250 | -0.005 | | 0.971 | -0.046 | | 0.765 | 0.178 | 0.212 |  |  |
| Whole Image | -0.217 | 0.115 | -0.200 | 0.155 | -0.026 | | 0.853 | 0.064 | | 0.651 | -0.168 | | 0.238 | -0.054 | | 0.704 | 0.169 | | 0.268 | 0.266 | 0.059 |  |  |
| **FAZ** | | | | | | | | | | | | | | | | | | | | | | | |
| FAZ-area(mm2) | 0.234 | 0.089 | 0.182 | 0.198 | -0.002 | | 0.987 | 0.173 | | 0.216 | 0.207 | | 0.144 | 0.010 | | 0.942 | 0.030 | | 0.845 | -0.011 | 0.937 |  |  |
| FAZ-circle(mm) | 0.233 | 0.091 | 0.130 | 0.357 | 0.055 | | 0.694 | 0.032 | | 0.820 | 0.119 | | 0.407 | 0.021 | | 0.882 | 0.090 | | 0.557 | -0.056 | 0.697 |  |  |
| **Optic disc** | | | | | | | | | | | | | | | | | | | | | | | |
| **SCP peripapillary VD(%)** | | | | | | | | | | | | | | | | | | | | | | | |
| 1.5mm Circle | -0.120 | 0.388 | -0.053 | 0.707 | 0.174 | | 0.213 | -0.020 | | 0.887 | 0.141 | | 0.322 | 0.112 | | 0.434 | 0.293 | | 0.051 | -0.069 | 0.631 |  |  |
| 2.5mm Circle | -0.130 | 0.349 | -0.195 | 0.167 | 0.029 | | 0.836 | -0.139 | | 0.321 | 0.034 | | 0.810 | 0.103 | | 0.470 | 0.349 | | **0.019** | -0.044 | 0.760 |  |  |
| 3.5mm Circle | -0.116 | 0.404 | -0.280 | **0.044** | -0.093 | | 0.509 | -0.091 | | 0.517 | -0.053 | | 0.709 | 0.089 | | 0.536 | 0.195 | | 0.200 | 0.004 | 0.980 |  |  |
| 5.0mm Circle | -0.067 | 0.632 | -0.280 | **0.044** | -0.172 | | 0.219 | -0.017 | | 0.903 | -0.020 | | 0.889 | 0.059 | | 0.680 | 0.025 | | 0.873 | 0.002 | 0.990 |  |  |
| **Inner Circle** | | | | | | | | | | | | | | | | | | | | | | | |
| Superior | -0.031 | 0.824 | -0.172 | 0.222 | 0.002 | | 0.989 | 0.018 | | 0.899 | 0.048 | | 0.740 | -0.044 | | 0.759 | 0.030 | | 0.845 | 0.094 | 0.510 |  |  |
| Nasal | -0.042 | 0.765 | -0.184 | 0.191 | 0.020 | | 0.886 | -0.124 | | 0.378 | -0.047 | | 0.742 | -0.173 | | 0.226 | 0.084 | | 0.581 | 0.168 | 0.238 |  |  |
| Inferior | -0.259 | 0.058 | -0.249 | 0.075 | 0.102 | | 0.467 | 0.087 | | 0.534 | 0.098 | | 0.495 | 0.160 | | 0.261 | 0.285 | | 0.057 | -0.156 | 0.274 |  |  |
| Temporal | -0.087 | 0.530 | -0.067 | 0.637 | -0.035 | | 0.802 | -0.057 | | 0.683 | -0.065 | | 0.653 | 0.138 | | 0.334 | 0.253 | | 0.094 | -0.002 | 0.987 |  |  |
| **Middle Circle** | | | | | | | | | | | | | | | | | | | | | | | |
| Superior | 0.074 | 0.596 | -0.098 | 0.488 | -0.271 | | 0.050 | 0.071 | | 0.616 | -0.069 | | 0.628 | -0.124 | | 0.385 | -0.027 | | 0.860 | 0.175 | 0.219 |  |  |
| Nasal | -0.001 | 0.991 | -0.253 | 0.070 | -0.188 | | 0.177 | -0.069 | | 0.623 | -0.255 | | 0.071 | -0.042 | | 0.769 | -0.088 | | 0.567 | 0.046 | 0.748 |  |  |
| Inferior | -0.053 | 0.705 | -0.325 | **0.019** | -0.196 | | 0.160 | -0.005 | | 0.971 | 0.134 | | 0.349 | 0.174 | | 0.222 | -0.096 | | 0.529 | -0.190 | 0.182 |  |  |
| Temporal | -0.100 | 0.473 | -0.201 | 0.154 | -0.107 | | 0.446 | -0.075 | | 0.594 | -0.131 | | 0.358 | -0.057 | | 0.694 | 0.129 | | 0.400 | 0.176 | 0.217 |  |  |
| **Outer Circle** | | | | | | | | | | | | | | | | | | | | | | | |
| Superior | 0.092 | 0.509 | -0.191 | 0.174 | -0.259 | | 0.061 | 0.172 | | 0.218 | -0.120 | | 0.402 | -0.032 | | 0.824 | -0.168 | | 0.269 | 0.042 | 0.767 |  |  |
| Nasal | 0.112 | 0.420 | -0.045 | 0.752 | -0.188 | | 0.177 | -0.139 | | 0.321 | 0.012 | | 0.932 | 0.216 | | 0.127 | -0.225 | | 0.137 | **-0.280** | **0.047** |  |  |
| Inferior | 0.031 | 0.823 | -0.309 | **0.026** | -0.264 | | 0.056 | 0.057 | | 0.686 | 0.058 | | 0.685 | 0.211 | | 0.137 | -0.082 | | 0.591 | -0.184 | 0.196 |  |  |
| Temporal | -0.151 | 0.275 | -0.095 | 0.502 | -0.044 | | 0.755 | 0.015 | | 0.916 | 0.013 | | 0.927 | -0.099 | | 0.490 | 0.054 | | 0.726 | 0.121 | 0.400 |  |  |
| Whole Image | -0.011 | 0.934 | -0.273 | 0.050 | -0.178 | | 0.203 | -0.011 | | 0.937 | -0.072 | | 0.617 | -0.024 | | 0.869 | 0.016 | | 0.919 | 0.092 | 0.519 |  |  |
| **DCP peripapillary VD(%)** | | | | | | | | | | | | | | | | | | | | | | | |
| 1.5mm Circle | -0.036 | 0.794 | -0.132 | 0.352 | 0.172 | | 0.217 | -0.107 | | 0.446 | 0.005 | | 0.973 | -0.100 | | 0.486 | 0.251 | | 0.097 | 0.227 | 0.109 |  |  |
| 2.5mm Circle | -0.146 | 0.292 | -0.235 | 0.094 | -0.008 | | 0.957 | -0.207 | | 0.138 | -0.096 | | 0.502 | 0.103 | | 0.471 | 0.015 | | 0.920 | 0.008 | 0.957 |  |  |
| 3.5mm Circle | -0.129 | 0.353 | -0.182 | 0.196 | -0.099 | | 0.480 | -0.116 | | 0.406 | 0.027 | | 0.850 | 0.192 | | 0.178 | -0.095 | | 0.535 | -0.116 | 0.416 |  |  |
| 5.0mm Circle | -0.126 | 0.365 | -0.122 | 0.387 | -0.164 | | 0.241 | -0.113 | | 0.422 | 0.031 | | 0.827 | 0.176 | | 0.216 | -0.140 | | 0.358 | -0.123 | 0.389 |  |  |
| **Inner Circle** | | | | | | | | | | | | | | | | | | | | | | | |
| Superior | -0.004 | 0.976 | -0.028 | 0.845 | -0.244 | | 0.078 | -0.054 | | 0.701 | 0.110 | | 0.440 | 0.177 | | 0.213 | -0.374 | | **0.011** | -0.138 | 0.336 |  |  |
| Nasal | -0.188 | 0.174 | -0.166 | 0.239 | -0.006 | | 0.965 | -0.088 | | 0.531 | 0.153 | | 0.284 | 0.120 | | 0.403 | -0.057 | | 0.712 | -0.120 | 0.401 |  |  |
| Inferior | -0.142 | 0.306 | -0.292 | **0.036** | -0.144 | | 0.304 | 0.086 | | 0.539 | 0.129 | | 0.368 | -0.002 | | 0.988 | -0.059 | | 0.700 | -0.036 | 0.799 |  |  |
| Temporal | -0.043 | 0.758 | -0.084 | 0.556 | -0.077 | | 0.585 | -0.037 | | 0.791 | -0.267 | | 0.059 | -0.017 | | 0.906 | -0.020 | | 0.896 | 0.160 | 0.261 |  |  |
| **Middle Circle** | | | | | | | | | | | | | | | | | | | | | | | |
| Superior | -0.016 | 0.909 | -0.153 | 0.278 | -0.185 | | 0.186 | -0.009 | | 0.948 | 0.050 | | 0.730 | 0.006 | | 0.965 | -0.098 | | 0.523 | 0.095 | 0.506 |  |  |
| Nasal | -0.058 | 0.676 | -0.085 | 0.551 | -0.212 | | 0.128 | -0.135 | | 0.333 | -0.120 | | 0.402 | 0.329 | | **0.018** | -0.226 | | 0.136 | -0.328 | **0.019** |  |  |
| Inferior | -0.016 | 0.910 | -0.063 | 0.658 | -0.142 | | 0.309 | -0.042 | | 0.766 | 0.215 | | 0.131 | 0.303 | | **0.031** | -0.338 | | **0.023** | -0.324 | **0.020** |  |  |
| Temporal | -0.030 | 0.831 | 0.018 | 0.898 | 0.066 | | 0.638 | 0.039 | | 0.782 | 0.018 | | 0.901 | -0.009 | | 0.949 | 0.054 | | 0.727 | 0.035 | 0.808 |  |  |
| **Outer Circle** | | | | | | | | | | | | | | | | | | | | | | | |
| Superior | -0.022 | 0.876 | -0.185 | 0.189 | -0.291 | | **0.035** | -0.002 | | 0.989 | -0.108 | | 0.451 | -0.031 | | 0.826 | -0.152 | | 0.318 | 0.090 | 0.529 |  |  |
| Nasal | -0.172 | 0.213 | 0.010 | 0.944 | -0.283 | | **0.040** | -0.120 | | 0.391 | 0.084 | | 0.557 | 0.130 | | 0.365 | -0.085 | | 0.580 | -0.127 | 0.374 |  |  |
| Inferior | -0.052 | 0.711 | -0.197 | 0.161 | -0.088 | | 0.532 | -0.027 | | 0.847 | 0.197 | | 0.166 | 0.274 | | 0.052 | -0.224 | | 0.139 | -0.258 | 0.067 |  |  |
| Temporal | 0.025 | 0.855 | 0.088 | 0.533 | 0.066 | | 0.640 | -0.038 | | 0.786 | -0.072 | | 0.617 | -0.034 | | 0.812 | -0.053 | | 0.731 | 0.021 | 0.884 |  |  |
| Whole Image | -0.091 | 0.512 | -0.080 | 0.573 | -0.197 | | 0.158 | -0.145 | | 0.300 | 0.015 | | 0.918 | 0.102 | | 0.475 | -0.121 | | 0.429 | -0.061 | 0.670 |  |  |

Notes: SLEDAI: systemic lupus erythematosus disease activity index; ESR: erythrocyte sedimentation rate; CRP: C-reactive protein; GFR: glomerular filtration rate; SCP: superficial capillary plexus; DCP: deep capillary plexus; VD: vessel density; VLD: vessel length density; FAZ: foveal avascular zone; Parafoveal: macular region 0.5–1.5 mm from the foveal center; Peripapillary: optic disc region surrounding the optic nerve head. P values below 0.05 indicates statistical signific

**Supplementary Table S3.** **Comparison of macular and optic disc vascular density and vessel length density among SLE patients with GC and without GC**

| **Variables Median (IQR)** | **Without GC (n = 13)** | | **With GC (n = 41)** | | **P** | |
| --- | --- | --- | --- | --- | --- | --- |
|  |  |  |  |  |  |  |
| **Macula** |  |  |  |  |  |  |
| **SCP parafoveal VD(%)** | | | | | |  |
| 1mm Circle | 22.0 (17.9, 23.2) | | 21.9 (18.3, 23.8) | | 0.693 | |
| 2.5mm Circle | 35.2 (33.6, 37.6) | | 36.3 (33.7, 38.0) | | 0.769 | |
| Loop | 37.7 (36.7, 41.0) | | 38.8 (36.4, 40.9) | | 0.785 | |
| Superior | 39.0 (36.7, 42.5) | | 39.0 (36.9, 40.7) | | 0.578 | |
| Nasal | 42.1 (34.8, 42.9) | | 40.0 (37.4, 41.4) | | 0.270 | |
| Inferior | 38.2 (35.1, 40.0) | | 39.1 (36.0, 40.6) | | 0.606 | |
| Temporal | 40.4 (34.3, 42.1) | | 39.8 (36.9, 41.8) | | 0.848 | |
| Whole Image | 37.5 (34.2, 39.7) | | 37.9 (35.7, 38.9) | | 0.976 | |
| **SCP parafoveal VLD(%)** | | | | | |  |
| 1mm Circle | 3.7 (3.0, 4.1) | | 3.8 (3.3, 4.2) | | 0.606 | |
| 2.5mm Circle | 6.0 (5.8, 6.6) | | 6.4 (5.9, 6.7) | | 0.564 | |
| Superior | 6.9 (6.1, 7.3) | | 6.7 (6.4, 7.1) | | 0.911 | |
| Nasal | 7.0 (6.1, 7.5) | | 6.9 (6.3, 7.3) | | 0.592 | |
| Inferior | 6.7 (6.0, 7.1) | | 6.7 (6.3, 7.1) | | 0.491 | |
| Temporal | 7.0 (6.0, 7.2) | | 7.0 (6.5, 7.3) | | 0.485 | |
| Whole Image | 6.4 (5.9, 7.0) | | 6.6 (6.1, 6.9) | | 0.800 | |
| **DCP parafoveal VD(%)** | | | | | |  |
| 1mm Circle | 15.3 (11.7, 20.4) | | 15.0 (11.6, 19.6) | | 0.911 | |
| 2.5mm Circle | 37.4 (36.4, 38.7) | | 36.8 (35.0, 38.0) | | 0.606 | |
| Loop | 41.4 (40.3, 43.0) | | 41.1 (39.3, 42.2) | | 0.649 | |
| Superior | 40.6 (39.3, 44.0) | | 42.0 (39.5, 43.5) | | 0.911 | |
| Nasal | 42.7 (34.8, 43.0) | | 39.7 (38.5, 42.2) | | 0.944 | |
| Inferior | 40.6 (36.2, 41.8) | | 40.2 (39.2, 42.2) | | 0.498 | |
| Temporal | 43.7 (41.0, 44.3) | | 41.0 (39.5, 42.9) | | 0.379 | |
| Whole Image | 39.7 (39.2, 40.5) | | 38.6 (37.4, 39.7) | | 0.307 | |
| **DCP parafoveal VLD(%)** | | | | | |  |
| 1mm Circle | 3.0 (2.4, 3.9) | | 2.9 (2.4, 3.9) | | 0.879 | |
| 2.5mm Circle | 6.6 (6.4, 6.9) | | 6.6 (6.3, 6.9) | | 0.769 | |
| Superior | 7.3 (6.9, 7.9) | | 7.4 (7.1, 7.8) | | 0.413 | |
| Nasal | 7.3 (6.3, 7.5) | | 7.1 (6.9, 7.5) | | 0.754 | |
| Inferior | 7.1 (6.5, 7.4) | | 7.2 (6.9, 7.6) | | 0.288 | |
| Temporal | 7.6 (7.3, 7.7) | | 7.3 (7.0, 7.8) | | 0.686 | |
| Whole Image | 7.1 (7.0, 7.2) | | 7.0 (6.8, 7.3) | | 0.960 | |
| **FAZ** | | | | | |  |
| FAZ-area(mm^2^) | 0.4 (0.3, 0.4) | | 0.3 (0.3, 0.5) | | 0.863 | |
| FAZ-circle(mm) | 3.8 (3.2, 4.4) | | 3.4 (3.0, 4.5) | | 0.848 | |
| **Optic disc** |  | |  |  |  |  |
| **SCP peripapillary VD(%)** | | | | | |  |
| 1.5mm Circle | 12.1 (8.5, 18.4) | | 12.5 (9.8, 15.8) | | 0.976 | |
| 2.5mm Circle | 22.3 (18.5, 24.5) | | 22.2 (19.0, 23.7) | | 0.769 | |
| 3.5mm Circle | 27.8 (24.3, 28.9) | | 27.0 (24.1, 28.0) | | 0.537 | |
| 5.0mm Circle | 30.3 (28.0, 31.5) | | 28.8 (25.9, 30.6) | | 0.261 | |
| **Inner Circle** |  | |  | |  | |
| Superior | 23.1 (21.4, 28.3) | | 26.3 (22.9, 28.4) | | 0.307 | |
| Nasal | 23.6 (20.3, 32.3) | | 29.3 (25.6, 32.2) | | 0.424 | |
| Inferior | 27.9 (21.8, 29.7) | | 24.9 (19.5, 29.2) | | 0.206 | |
| Temporal | 26.4 (22.6, 34.1) | | 29.7 (25.3, 35.6) | | 0.485 | |
| **Middle Circle** |  | |  | |  | |
| Superior | 29.6 (28.4, 32.6) | | 31.2 (28.0, 33.6) | | 0.911 | |
| Nasal | 32.9 (30.5, 36.0) | | 31.8 (26.7, 35.8) | | 0.213 | |
| Inferior | 32.5 (31.3, 32.6) | | 30.1 (27.1, 32.6) | | **0.042** | |
| Temporal | 34.6 (31.2, 38.3) | | 34.5 (31.7, 35.8) | | 0.927 | |
| **Outer Circle** |  | |  | |  | |
| Superior | 33.2 (32.5, 34.4) | | 31.3 (27.3, 35.0) | | 0.192 | |
| Nasal | 32.8 (29.7, 35.0) | | 30.5 (26.9, 34.0) | | 0.142 | |
| Inferior | 33.2 (32.2, 35.2) | | 31.4 (28.1, 34.5) | | 0.117 | |
| Temporal | 31.0 (29.7, 35.9) | | 33.0 (28.6, 36.1) | | 0.708 | |
| Whole Image | 31.3 (29.5, 32.6) | | 29.7 (27.4, 31.3) | | 0.127 | |
| **DCP peripapillary VD(%)** | | | | | |  |
| 1.5mm Circle | 18.5 (13.4, 27.7) | | 17.2 (12.5, 25.7) | | 0.578 | |
| 2.5mm Circle | 20.0 (18.6, 20.7) | | 19.9 (18.3, 21.2) | | 0.754 | |
| 3.5mm Circle | 23.5 (21.6, 24.0) | | 22.8 (20.4, 24.4) | | 0.551 | |
| 5.0mm Circle | 24.6 (23.6, 25.9) | | 24.5 (22.1, 26.0) | | 0.649 | |
| **Inner Circle** |  | |  | |  | |
| Superior | 22.7 (19.0, 24.5) | | 21.2 (19.2, 23.3) | | 0.739 | |
| Nasal | 25.3 (19.6, 29.7) | | 22.7 (18.2, 26.2) | | 0.270 | |
| Inferior | 23.5 (20.1, 25.3) | | 23.1 (18.9, 25.9) | | 0.848 | |
| Temporal | 27.7 (22.1, 30.0) | | 26.7 (24.3, 30.3) | | 0.976 | |
| **Middle Circle** |  | |  | |  | |
| Superior | 23.5 (22.4, 26.0) | | 24.3 (21.8, 26.1) | | 0.976 | |
| Nasal | 29.3 (28.0, 31.0) | | 26.1 (22.0, 30.9) | | 0.087 | |
| Inferior | 25.9 (22.5, 28.2) | | 24.7 (22.1, 27.0) | | 0.551 | |
| Temporal | 26.1 (24.4, 29.4) | | 26.9 (23.9, 30.1) | | 0.746 | |
| **Outer Circle** |  | |  | |  | |
| Superior | 25.3 (24.7, 27.3) | | 25.6 (22.6, 27.6) | | 0.723 | |
| Nasal | 29.1 (27.0, 30.4) | | 30.2 (26.2, 33.4) | | 0.537 | |
| Inferior | 25.8 (23.5, 28.1) | | 25.2 (22.1, 26.9) | | 0.237 | |
| Temporal | 25.4 (22.8, 27.6) | | 24.7 (20.0, 30.1) | | 0.944 | |
| Whole Image | 26.7 (24.1, 27.6) | | 25.2 (23.1, 28.5) | | 0.693 | |

Notes: SCP: superficial capillary plexus; DCP: deep capillary plexus; VD: vessel density; VLD: vessel length density; FAZ: foveal avascular zone; P values below 0.05 indicates statistical significance.

**Supplementary Table S4.** **Comparison of macular and optic disc vascular density and vessel length density among SLE patients with Mycophenolate mofetil and without Mycophenolate mofetil**

| **Variables Median (IQR)** | **Without MMF (n = 34)** | | **With MMF (n = 20)** | | **P** | |
| --- | --- | --- | --- | --- | --- | --- |
|  |  |  |  |  |  |  |
| **Macula** |  |  |  |  |  |  |
| **SCP parafoveal VD(%)** | | | | | |  |
| 1mm Circle | 21.6 (18.5, 23.0) | | 23.4 (15.5, 24.0) | | 0.210 | |
| 2.5mm Circle | 36.5 (33.9, 38.1) | | 35.6 (33.5, 37.0) | | 0.203 | |
| Loop | 39.6 (37.4, 41.3) | | 38.1 (36.2, 39.4) | | 0.065 | |
| Superior | 39.3 (38.0, 42.3) | | 37.4 (36.6, 40.2) | | 0.079 | |
| Nasal | 40.4 (36.7, 41.7) | | 39.5 (36.7, 40.9) | | 0.342 | |
| Inferior | 39.2 (35.9, 40.6) | | 38.3 (34.5, 39.6) | | 0.325 | |
| Temporal | 40.6 (38.8, 42.5) | | 37.2 (36.0, 40.6) | | **0.020** | |
| Whole Image | 38.3 (36.2, 39.1) | | 36.9 (34.9, 38.1) | | 0.058 | |
| **SCP parafoveal VLD(%)** | | | | | |  |
| 1mm Circle | 3.7 (3.2, 4.0) | | 4.0 (2.8, 4.2) | | 0.271 | |
| 2.5mm Circle | 6.4 (5.8, 6.7) | | 6.2 (5.9, 6.5) | | 0.316 | |
| Superior | 6.9 (6.5, 7.3) | | 6.5 (6.1, 7.0) | | 0.179 | |
| Nasal | 7.0 (6.2, 7.4) | | 6.9 (6.3, 7.2) | | 0.420 | |
| Inferior | 6.8 (6.2, 7.1) | | 6.6 (5.9, 7.0) | | 0.338 | |
| Temporal | 7.1 (6.7, 7.4) | | 6.5 (6.2, 7.1) | | **0.045** | |
| Whole Image | 6.7 (6.2, 6.9) | | 6.3 (6.1, 6.7) | | 0.115 | |
| **DCP parafoveal VD(%)** | | | | | |  |
| 1mm Circle | 13.6 (11.4, 19.4) | | 16.6 (12.3, 19.9) | | 0.267 | |
| 2.5mm Circle | 36.7 (34.5, 38.6) | | 37.4 (35.5, 38.3) | | 0.316 | |
| Loop | 41.1 (39.3, 42.2) | | 41.2 (39.5, 42.3) | | 0.734 | |
| Superior | 41.7 (39.3, 43.7) | | 42.0 (39.3, 42.9) | | 0.865 | |
| Nasal | 39.6 (38.1, 42.5) | | 40.6 (38.0, 44.3) | | 0.420 | |
| Inferior | 40.2 (38.5, 41.7) | | 41.4 (39.3, 42.6) | | 0.237 | |
| Temporal | 41.5 (39.7, 44.6) | | 40.9 (39.5, 42.3) | | 0.307 | |
| Whole Image | 38.9 (37.2, 40.4) | | 39.0 (37.9, 39.7) | | 0.957 | |
| **DCP parafoveal VLD(%)** | | | | | |  |
| 1mm Circle | 2.8 (2.3, 3.8) | | 3.4 (2.5, 4.1) | | 0.173 | |
| 2.5mm Circle | 6.5 (6.2, 6.9) | | 6.7 (6.4, 6.9) | | 0.197 | |
| Superior | 7.3 (7.0, 7.9) | | 7.4 (7.1, 7.8) | | 0.774 | |
| Nasal | 7.1 (6.7, 7.4) | | 7.2 (6.8, 7.8) | | 0.352 | |
| Inferior | 7.2 (6.8, 7.4) | | 7.4 (7.1, 7.7) | | 0.111 | |
| Temporal | 7.4 (7.1, 7.8) | | 7.4 (7.2, 7.5) | | 0.661 | |
| Whole Image | 7.0 (6.7, 7.2) | | 7.1 (6.9, 7.3) | | 0.542 | |
| **FAZ** | | | | | |  |
| FAZ-area(mm^2^) | 0.4 (0.3, 0.5) | | 0.3 (0.3, 0.5) | | 0.543 | |
| FAZ-circle(mm) | 3.6 (3.1, 4.6) | | 3.3 (3.0, 3.8) | | 0.267 | |
| **Optic disc** |  | |  |  |  |  |
| **SCP peripapillary VD(%)** | | | | | |  |
| 1.5mm Circle | 12.5 (8.7, 15.7) | | 12.4 (10.1, 16.5) | | 0.554 | |
| 2.5mm Circle | 21.6 (18.0, 24.4) | | 22.5 (21.1, 23.5) | | 0.370 | |
| 3.5mm Circle | 26.6 (23.9, 28.2) | | 27.4 (25.7, 28.9) | | 0.463 | |
| 5.0mm Circle | 29.0 (26.1, 31.2) | | 30.2 (27.8, 31.5) | | 0.543 | |
| **Inner Circle** |  | |  | |  | |
| Superior | 26.0 (21.5, 28.3) | | 25.4 (23.2, 28.8) | | 0.507 | |
| Nasal | 27.9 (21.4, 32.1) | | 28.8 (25.8, 32.6) | | 0.420 | |
| Inferior | 25.6 (20.8, 29.4) | | 25.1 (19.6, 29.5) | | 0.943 | |
| Temporal | 28.7 (23.0, 34.3) | | 29.2 (24.7, 36.8) | | 0.788 | |
| **Middle Circle** |  | |  | |  | |
| Superior | 30.6 (27.9, 33.0) | | 31.7 (28.8, 34.5) | | 0.252 | |
| Nasal | 31.6 (27.6, 35.4) | | 32.0 (28.0, 36.1) | | 0.774 | |
| Inferior | 31.4 (29.3, 32.6) | | 28.7 (27.0, 32.8) | | 0.287 | |
| Temporal | 34.7 (31.3, 37.9) | | 34.5 (31.7, 36.7) | | 0.707 | |
| **Outer Circle** |  | |  | |  | |
| Superior | 32.6 (30.3, 34.3) | | 33.1 (27.2, 35.4) | | 0.971 | |
| Nasal | 30.2 (27.7, 33.4) | | 32.1 (27.2, 35.0) | | 0.361 | |
| Inferior | 31.8 (29.2, 34.8) | | 31.5 (29.5, 34.9) | | 0.900 | |
| Temporal | 32.5 (29.7, 35.8) | | 33.5 (28.6, 36.4) | | 0.667 | |
| Whole Image | 29.8 (27.5, 31.4) | | 30.1 (28.1, 32.6) | | 0.694 | |
| **DCP peripapillary VD(%)** | | | | | |  |
| 1.5mm Circle | 18.5 (12.6, 25.3) | | 16.2 (12.0, 28.3) | | 0.667 | |
| 2.5mm Circle | 20.0 (19.2, 21.3) | | 18.9 (16.9, 20.7) | | **0.041** | |
| 3.5mm Circle | 23.3 (21.4, 24.2) | | 22.6 (20.2, 24.1) | | 0.390 | |
| 5.0mm Circle | 24.5 (22.6, 25.9) | | 24.6 (22.7, 26.1) | | 0.816 | |
| **Inner Circle** |  | |  | |  | |
| Superior | 22.8 (19.6, 24.8) | | 20.2 (17.6, 22.2) | | 0.055 | |
| Nasal | 22.7 (19.2, 25.7) | | 23.6 (17.7, 27.7) | | 0.957 | |
| Inferior | 23.0 (19.9, 26.0) | | 23.1 (17.2, 25.2) | | 0.275 | |
| Temporal | 28.3 (25.5, 30.8) | | 24.4 (20.4, 27.2) | | **0.012** | |
| **Middle Circle** |  | |  | |  | |
| Superior | 23.8 (21.7, 25.5) | | 24.3 (22.8, 26.9) | | 0.361 | |
| Nasal | 28.0 (23.6, 31.0) | | 28.0 (23.1, 31.1) | | 0.914 | |
| Inferior | 25.9 (22.5, 28.0) | | 24.3 (21.6, 26.2) | | 0.275 | |
| Temporal | 27.2 (23.2, 29.7) | | 26.7 (24.8, 30.4) | | 0.525 | |
| **Outer Circle** |  | |  | |  | |
| Superior | 25.2 (22.7, 27.2) | | 26.0 (23.7, 27.7) | | 0.554 | |
| Nasal | 28.7 (26.3, 31.2) | | 32.0 (29.1, 35.2) | | **0.013** | |
| Inferior | 25.5 (22.2, 27.2) | | 25.2 (22.7, 26.4) | | 0.830 | |
| Temporal | 24.4 (22.2, 29.6) | | 25.8 (18.7, 30.0) | | 0.943 | |
| Whole Image | 25.7 (23.8, 27.5) | | 25.2 (23.7, 28.5) | | 0.554 | |

Notes: SCP: superficial capillary plexus; DCP: deep capillary plexus; VD: vessel density;

VLD: vessel length density; FAZ: foveal avascular zone; P values below 0.05 indicates statistical significance

**Supplementary Table S5.** Univariate and multivariate linear regression analysis of the impact of Belimumab and other factors on DCP parafoveal VD

| **Variable** | **Univariate Analysis** | |  | **Multivariate Analysis** | |
| --- | --- | --- | --- | --- | --- |
|  | **β（95%CI）** | **P** |  | **β（95%CI）** | **P** |
| Disease Duration, year | -0.01(-0.201,0.191) | 0.964 |  |  |  |
| LN(1vs0) | -2.04 (-4.39,0.3) | 0.086 |  | -1.4 (-3.81~1) | 0.258 |
| Belimumab(1vs0) | -2.29(-4.55,-0.02) | **0.048** |  | -1.82(-4.17~0.53) | 0.135 |
| HCQ(1vs0) | 0.77(-1.75,3.29) | 0.542 |  |  |  |
| MMF(1vs0) | -1.93 (-4.3,0.45) | 0.11 |  |  |  |

Notes: LN, lupus nephritis; HCQ, hydroxychloroquine; MMF, mycophenolate mofeti; CI, confidence interval; P values below 0.05 indicate statistical significance.

**Supplementary Table S6. Comparison of baseline levels between the patients receiving and not receiving Belimumab treatment**

| **Variables**  **Median (IQR)** | **Without Belimumab**  **(n = 27)** | **With Belimumab**  **(n = 27)** | **p** |
| --- | --- | --- | --- |
| Age(Years), Mean ± SD | 35.76 ± 11.22 | 32.88 ±10.33 | 0.370 |
| Disease duration(year) | 1.50 (1.00, 10.00) | 1.50 (0.00, 6.00) | **0.044** |
| SLEDAI score | 12.00 (8.00, 14.00) | 14.00 (11.00, 17.00) | 0.169 |
| Anti-ds-DNA  antibody positivity,n(%) | 16 (61.5) | 19 (70.4) | 0.497 |
| ESR | 40.00 (15.25, 82.25) | 35.00 (14.00, 68.50) | 0.466 |
| CRP | 3.93 (0.80, 8.89) | 3.42 (0.97, 19.32) | 0.631 |
| GFR | 109.05 (99.19, 128.05) | 106.22 (91.24, 118.36) | 0.534 |
| C3 | 0.86 (0.66, 0.98) | 0.62 (0.33, 0.95) | 0.052 |
| C4 | 0.16 (0.09, 0.22) | 0.12 (0.06, 0.21) | 0.504 |
| 24hUpro | 135.00 (94.00, 462.75) | 224.00 (113.50, 723.50) | 0.199 |
| Albumin | 37.15 (33.15, 38.80) | 32.70 (28.10, 36.70) | 0.064 |
| Creatinine | 58.00 (51.75, 64.75) | 62.00 (50.00, 65.00) | 0.888 |
| LN, n (%) | 6 (22.2) | 15 (55.6) | **0.012** |
| Type of LN, n (%) |  |  | 0.244 |
| I | 0 (0) | 1 (9.1) |  |
| III+V | 0 (0) | 1 (9.1) |  |
| IV | 0 (0) | 4 (36.4) |  |
| IV+V | 4 (80) | 5 (45.5) |  |
| V | 1 (20) | 0 (0) |  |

Notes:CRP: C-reactive protein; ESR: erythrocyte sedimentation rate; GFR: glomerular filtration rate; C3: complement component 3; C4: complement component 4; 24hUpro: 24-hour urinary protein; SLEDAI: systemic lupus erythematosus disease activity index; LN: lupus nephritis. P values below 0.05 indicates statistical significance.
